# The Effects of Sub-Regional Climate Velocity on the Distribution and Spatial Extent of Marine Species Assemblages

**DOI:** 10.1371/journal.pone.0149220

**Published:** 2016-02-22

**Authors:** Kristin M. Kleisner, Michael J. Fogarty, Sally McGee, Analie Barnett, Paula Fratantoni, Jennifer Greene, Jonathan A. Hare, Sean M. Lucey, Christopher McGuire, Jay Odell, Vincent S. Saba, Laurel Smith, Katherine J. Weaver, Malin L. Pinsky

**Affiliations:** 1 National Oceanic and Atmospheric Administration (NOAA), Northeast Fisheries Science Center, Woods Hole, Massachusetts, United States of America; 2 The Nature Conservancy, Boston, Massachusetts, United States of America; 3 National Oceanic and Atmospheric Administration (NOAA), Northeast Fisheries Science Center, Narragansett, Rhode Island, United States of America; 4 National Oceanic and Atmospheric Administration (NOAA), Northeast Fisheries Science Center, c/o Geophysical Fluid Dynamics Laboratory, Princeton University Forrestal Campus, Princeton, New Jersey, United States of America; 5 Dept. of Ecology, Evolution, and Natural Resources, Rutgers University, New Brunswick, New Jersey, United States of America; Bangor University, UNITED KINGDOM

## Abstract

Many studies illustrate variable patterns in individual species distribution shifts in response to changing temperature. However, an assemblage, a group of species that shares a common environmental niche, will likely exhibit similar responses to climate changes, and these community-level responses may have significant implications for ecosystem function. Therefore, we examine the relationship between observed shifts of species in assemblages and regional climate velocity (i.e., the rate and direction of change of temperature isotherms). The assemblages are defined in two sub-regions of the U.S. Northeast Shelf that have heterogeneous oceanography and bathymetry using four decades of bottom trawl survey data and we explore temporal changes in distribution, spatial range extent, thermal habitat area, and biomass, within assemblages. These sub-regional analyses allow the dissection of the relative roles of regional climate velocity and local physiography in shaping observed distribution shifts. We find that assemblages of species associated with shallower, warmer waters tend to shift west-southwest and to shallower waters over time, possibly towards cooler temperatures in the semi-enclosed Gulf of Maine, while species assemblages associated with relatively cooler and deeper waters shift deeper, but with little latitudinal change. Conversely, species assemblages associated with warmer and shallower water on the broad, shallow continental shelf from the Mid-Atlantic Bight to Georges Bank shift strongly northeast along latitudinal gradients with little change in depth. Shifts in depth among the southern species associated with deeper and cooler waters are more variable, although predominantly shifts are toward deeper waters. In addition, spatial expansion and contraction of species assemblages in each region corresponds to the area of suitable thermal habitat, but is inversely related to assemblage biomass. This suggests that assemblage distribution shifts in conjunction with expansion or contraction of thermal habitat acts to compress or stretch marine species assemblages, which may respectively amplify or dilute species interactions to an extent that is rarely considered. Overall, regional differences in climate change effects on the movement and extent of species assemblages hold important implications for management, mitigation, and adaptation on the U.S. Northeast Shelf.

## Introduction

The assessment and prediction of climate change effects on biota is a major area of research [1–7]. Most studies examine the climate-related responses of individual species and illustrate variable patterns in species distribution shifts in response to drivers such as changing temperature [8–12] and fishing pressure [5, 13–16]. However, interactions between species and aggregate community-level responses may have important implications for ecosystem functioning [17]. Additionally, as geography and environmental conditions vary over a particular landscape, one might expect that species with similar bathy-thermal preferences or restrictions to particular environments will respond to climate change in comparable ways [3]. Therefore, understanding the temporal and spatial persistence of species assemblages—co-occurring groups of species identified by common environmental characteristics—may help identify unique ecological qualities that describe the condition of a collection of species and the implications of climate change at the community level.

Of particular interest is the question of whether species-level responses to climate change are generalizable among taxonomic groups [17] and across regions [18]. Taxonomically, some studies have shown consistency in community-level phenological responses [19], patterns of ecotypic variation across groups of species [20], and community distribution and composition [21]. However, these studies have generally focused on terrestrial biota. In the ocean, community-level climate responses may be more pronounced, given that marine ectotherms can more fully colonize and utilize the range of latitudes that offer tolerable thermal conditions than their terrestrial counterparts [22]. Spatially, species responses to climatic change are quite variable. Some studies have illustrated that certain species exhibit poleward shifts and/or movements to deeper waters or higher elevations on land [4, 6, 7, 15, 16, 23, 24], while others have shown that the direction of species shifts can vary regionally due to sub-optimal environmental conditions or habitat, physiographic constraints (e.g., land barriers), or an inability to colonize new regions [18]. Similarities in regional climate responses within marine assemblages defined by bathy-thermal preferences have not been clearly demonstrated before. However, if they are present, these differences may hold important implications for species interactions such as predator-prey dynamics and competition as species shift into new areas and undergo range expansion or contraction. Additionally, regional differences in climate responses may have management implications as new species enter or vacate traditional habitat. For example, increasingly concentrated species distributions may result in increased vulnerability to capture by fisheries [25].

**Fig 1:**
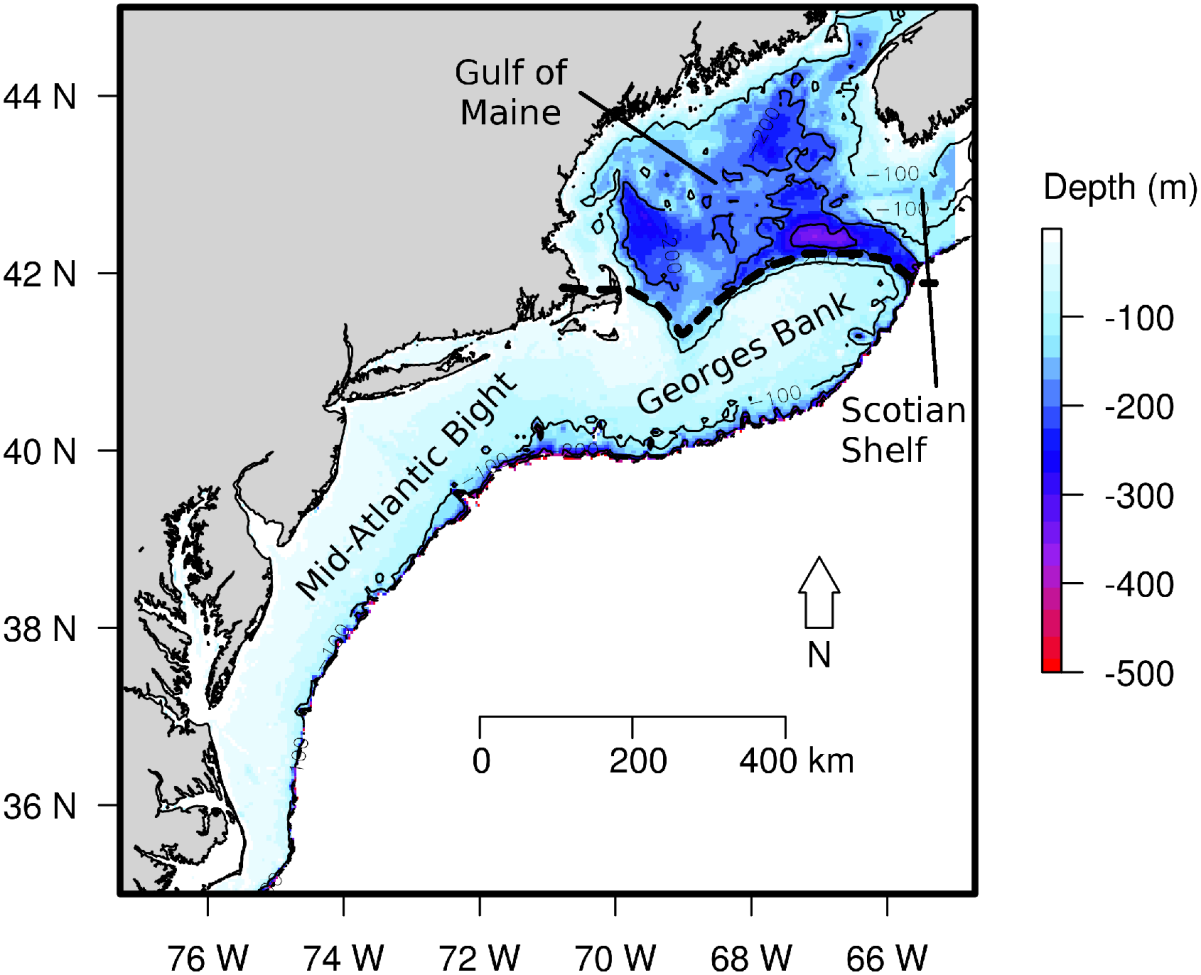
Study area. The Northeast U.S. Shelf illustrating the southern region: the Mid-Atlantic Bight and Georges Bank, and northern region: the Gulf of Maine with shaded bathymetry (meters depth).

The concept that marine species track climate velocities, the rate and direction of change of temperature isotherms [18], has been used with success to explain climate responses at the individual species level. Here, we apply this concept to marine species assemblages on the U.S. Northeast Shelf (NES) to understand how groups of species that are experiencing similar oceanographic and bathymetric conditions will respond to climate change in terms of latitudinal- and depth-related directions and rates of shift. The NES, a region with highly variable oceanography and geography along a latitudinal gradient, provides an opportunity to understand the role of sub-regional scale processes and constraints on the scope for assemblage-level distributional shifts in relation to climate velocity. Within the NES, the Gulf of Maine in the north (Fig 1) is a semi-enclosed continental shelf sea with deep and variable topography. Its coastline may constrain the potential range and direction of movement, possibly driving species to seek preferable water temperatures in deeper waters. Conversely, the more uniformly shallow bathymetry on Georges Bank and along the Mid-Atlantic Bight in the southern NES (Fig 1) may place far fewer constraints on latitudinal movement. Past studies illustrated differences in assemblage composition between the northern and southern NES [5] and that species in general were shifting in a predominantly northeast direction over the whole U.S. NES region in response to climate change [18]. Here, we build on these studies and posit that strong local physiographic constraints can result in regional differences in the local climate-related responses of species assemblages in terms of direction and rate of spatial shifts. Additionally, we posit that, within assemblages, species will have comparable climate-related responses in terms of direction and rate of shift [8, 15].

As marine species assemblages shift regionally, interactions (e.g., competition, predator-prey relationships) between individual species within and between the assemblages are likely to be affected [26, 27]. One potential result of shifts in species assemblages in response to climate change is that the range distribution of the assemblages will expand or contract given the presence, or lack of, habitable areas into which to shift. If species assemblages are compressed into smaller areas, then the probability of enhanced interactions is likely greater, and more so if the biomass of the species assemblage increases [28]. The converse is also probably true. Whereas we might expect the distribution range and the abundance of a given species to be related under MacCall’s basin hypothesis [29, 30], which postulates that a species’ geographic range is correlated with population size and is a function of habitat selection (i.e., larger populations will inhabit larger spatial extents), the same expectation may not hold at the assemblage level due to changes in the frequency of species interactions. To explore the potential for enhanced or reduced species interactions within species assemblages, we compare changes in the range size and biomass of the assemblages over time. Increases in biomass coupled with a contraction in range extent for a given assemblage may suggest enhanced species interactions within an assemblage.

The analyses presented here are intended to explore climate change responses at the community level. Therefore, we define groups of species occupying similar bathy-thermal niches in two areas of the NES that have strong differences in bathymetry and oceanography and investigate the role of sub-regional scale processes and constraints on the scope for assemblage-level distributional shifts in relation to climate velocity. We explore whether latitudinal and depth-related climate responses between assemblages are consistent in terms of direction and rate of shift and discuss how species within assemblages are shifting relative to climate-related oceanic changes. Finally, we characterize changes in the spatial extent and biomass of the assemblages over time and discuss potential implications for these patterns on species interactions within the assemblages.

## Methods

### Survey data

Four species clusters were identified using data from the Northeast Fisheries Science Center (NEFSC) spring (February-April) and fall (September-October) bottom trawl survey in the Gulf of Maine (northern NES) and Mid-Atlantic Bight/Georges Bank (southern NES) from 1968–2012 based on species biomass and environmental variables sampled at individual trawl locations. In order to eliminate rare species or species that were not frequently sampled, species caught in fewer than 10 years were removed. Annual survey coverage was consistent over time (S1 and S2 Figs, for spring and fall respectively).

### Data analysis

K-means [31] and hierarchical clustering [24] using complete linkage were utilized to distinguish species clusters based on NEFSC bottom trawl surveys in each region. Using multiple methods to evaluate the robustness of groupings defined by clustering routines, which have been labeled as subjective, is recommended to improve statistical power [32, 33]. For both methods, species were clustered by abundance-weighted mean depth, surface, and bottom temperature. Only oceanographic variables sampled on the bottom trawl surveys were included in the analysis. Bottom and surface salinity were tested as additional clustering variables, but did not have a significant impact on the assemblages and therefore were not included. Latitude/longitude were not used as clustering metrics so as to objectively identify species assemblages based on ‘realized bathy-thermal niches’ without a priori knowledge of geographic proximity. All statistical analyses were run in R 3.0.2 [34] using the *k-means* and *hclust* functions in the ‘stats’ package. Four species assemblages were considered optimal based on three measures from the ‘clValid’ package in R [35]: (1) connectedness (a measure of the frequency with which observations are placed in the same cluster as their nearest neighbors [36], (2) compactness (a measure of the homogeneity of the clusters) [37], and (3) silhouette width (the average of each observation’s clustering confidence value) [38].

To assess potential shifts in species assemblage distribution over time, the time series was divided into four, roughly decadal, periods in each season: 1968–1978, 1979–1989, 1990–2000, and 2001–2012, and assemblages were defined in each period. Additionally, because the topography and oceanography in the northern NES is vastly different from the southern NES, species assemblages were evaluated separately by region. For each assemblage, ‘principal’ species were defined as those that grouped together in both methods, in at least three of four periods.

### Directionality and movement of species clusters

To examine changes in distribution relative to climate conditions, centers of biomass for each species in five-year time blocks from 1968–2012 were calculated following an approach developed by colleagues [5], which re-grids latitude and longitude using along-shelf and cross-shelf positions to avoid centers of biomass outside the survey area. The re-gridded points were weighted by the biomass at each point and averaged to determine the centers of biomass for each species in a region. The bearing and direction of straight-line distance between the centers of abundance in the first and the last period was calculated for each species in each season and region using the *bearing* and *distHaversine* functions of the ‘geosphere’ package in R [39] providing an indication of species distribution changes over the time series. Rayleigh’s test of uniformity was used to assess the uniformity of the mean resultant bearing within each regional assemblage [40], i.e., to test for the existence of a modal direction of shift across the species in an assemblage.

### Observed species shifts versus climate velocity

Using the re-gridded latitude/longitude positions and depths at each survey location, we calculated observed distribution shifts based on mean latitude and depth and compared these to species-specific climate velocities, i.e., the predicted distribution shift of a species given temperature changes over time. Adapting an approach presented by colleagues [18], we calculated centroids of biomass in five year time blocks defined as biomass-weighted average latitude and biomass-weighted depth. We regressed the latitude and depth-based centroids against the five-year time periods to measure the average rate of species distribution shift. To calculate species-specific climate velocities, we estimated thermal envelopes for each species by fitting Generalized Additive Models (GAMs) to the survey biomass data. Because trawl survey data are subject to many zero observations, we used delta-lognormal GAMs [41], which model presence-absence separately from logged positive observations. The independent variables were surface and bottom temperature, fit with penalized regression splines, and habitat stratum and mean annual abundance from the survey. Stratum accounts for differences in regional habitat quality and mean annual abundance accounts for region-wide changes in abundance due to factors other than climate change [18], e.g., fishing pressure.

The predictions from the GAMs were used as weights in calculating centroids of thermal envelopes in each five-year time block. The centroids of the thermal envelopes were defined as prediction-weighted average latitude and depth. Rates and directions of latitudinal and depth-related shifts of thermal envelope centroids were determined similarly to the observed species shifts by regressing the latitude or depth of the centroid against the five-year period. Slopes of the regressions were °N/yr or m/yr and represented taxon-specific climate velocities. Kruskal-Wallis rank sum tests were used to determine statistically significant differences in the climate velocities between assemblages [42].

To explore whether the latitudinal regression models are biased due to constraints imposed by the limits of the sampling region, we also applied truncated regression models, using a southern or left truncation in the northern NES and a northern or right truncation in the southern NES, i.e., in the dominant direction of species shift. Details are presented in S1 Appendix and results are highlighted in the text.

### The role of thermal habitat versus biomass in determining assemblage area

To explore the spatial distribution and area of the species assemblages, we calculated the kernel densities of both the assemblage locations and the predicted thermal habitat (from the GAM predictions). We determined ‘core’ kernel areas as those locations where the overall assemblage biomass was greater than one standard deviation above the mean and created polygons for each core kernel for each assemblage, season, region, and five-year time block. We then computed the area of these core kernel areas using the *gArea* function from the ‘rgeos’ package in R [34]. We also computed the total biomass of a given assemblage in each region, season, and five-year time block. To test whether there is a relationship between the assemblage range area and either the biomass of an assemblage or the thermal habitat area of an assemblage we fit a linear mixed effects model with the thermal habitat area and cluster biomass in a given region (*i*), and season (*j*) as fixed variables and included ‘region/season’ as a nested random variable:

$$\text{Kernel Area = }\alpha +\beta_{1}\cdot {\text{Thermal Area}}_{ij}+\beta_{2}\cdot {\text{Biomass}}_{ij}+a_{i}+\varepsilon_{ij}$$

We followed a ten-step protocol to determining optimal model structure [43].

## Results

**Fig 2:**
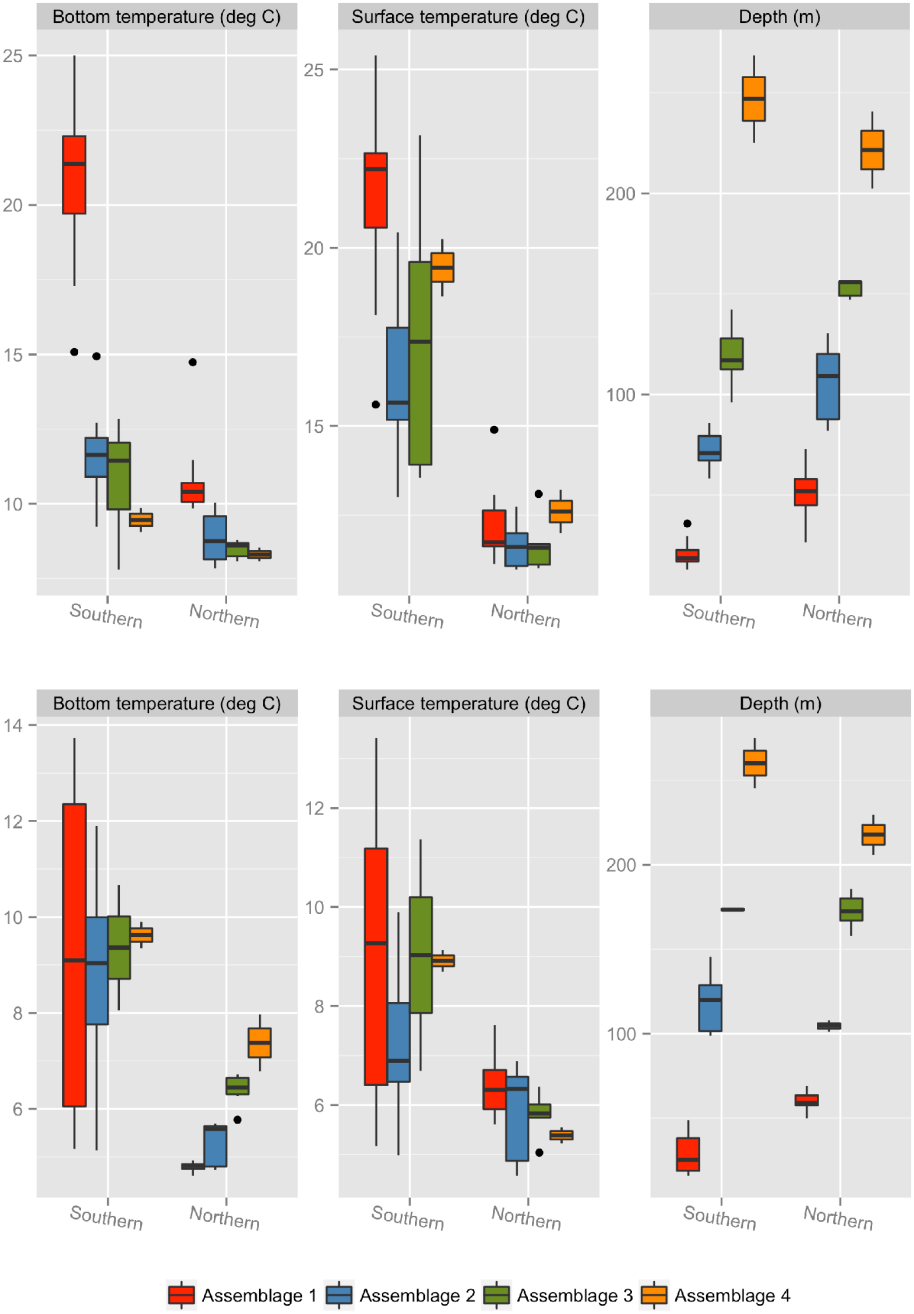
Assemblage characteristics. Boxplots of surface temperature, bottom temperature, and depth for each of the core species clusters in the Gulf of Maine (northern NES) or Mid-Atlantic Bight (southern NES) sampled during the NEFSC fall (top panels) and spring (bottom panels) bottom trawl surveys. Clusters in each region comprise different species, but are labeled 1 through 4 based on an increasing depth scale in each season and region.

The species assemblages defined in environmental space (i.e., based on temperature and depth) specify the realized bathy-thermal niches of a collection of species. The NES demersal species group into four spatially distinct assemblages per region and season (Fig 2 and S3 Fig; S1 Table) characterized by ‘core species’ that persistently cluster together over time in the fall (Table 1) and spring (Table 2).

**Table 1:** Description of ‘core’ species (i.e., identified in similar clusters by both k-means and hierarchical clustering and in at least three out of four periods) found in each species assemblage defined for the northern and southern regions of the fall bottom trawl survey.

**North: Gulf of Maine**	
**Assemblage**	**Description**
**1**: Alewife, American lobster, Atlantic mackerel, Blueback herring, Little skate, Longfin squid, Scup, Windowpane flounder, Winter flounder, Winter skate, Yellowtail flounder	Mid-trophic level species; mainly demersal; primarily in shallower, coastal inshore waters/protected bays and estuaries; some spawn in estuaries and rivers.
**2**: American plaice, American shad, Haddock, Red hake, Spiny dogfish, Wolffish	Mix of mid- and higher trophic level species; mix of pelagic and demersal species; found more frequently in coastal inshore waters.
**3**: Barndoor skate, Monkfish, Silver hake, White hake, Witch flounder	Mainly higher trophic level species; generally demersal, mid to deep waters and occasionally/soft bottom.
**4**: Blackbelly rosefish, Smooth skate	Mid-trophic level species; bathydemersal, found in very deep waters.
**South: Mid-Atlantic Bight/Georges Bank**	
**1**: Atlantic croaker, Banded drum, Black sea bass, Bluefish, Bluntnose stingray, Bullnose stingray, Clearnose skate, Cownose ray, Cravelle jack, Northern kingfish, Northern puffer, Northern sea robin, Pig fish, Pin fish, Roughtail stingray, Scup, Sharpnose shark, Smooth dogfish, Southern stingray, Spiny butterfly ray, Spot, Striped burrfish, Striped sea robin, Summer flounder, Tautog, Weakfish, Windowpane flounder	Mix of mid- and higher trophic levels; mainly demersal and reef-associated; strongly tied to coastal inshore waters, bays, estuaries, etc. and warm waters.
**2**: Alewife, American shad, Atlantic cod, Atlantic herring, Barndoor skate, Butterfish, Fourspot flounder, Longhorn sculpin, Red hake, Sea raven, Sea scallop, Silver hake, Spiny dogfish, Yellowtail flounder	Mix of mid- and higher trophic level species; mainly demersal/benthic; Generally in coastal waters, or have the ability to migrate between deeper and shallower depths.
**3**: American lobster, American plaice, Monkfish, Rosette skate, Shortfin squid, Thorney skate, White hake	Mid- to high trophic levels; Found mostly around shelf edge over soft bottoms.
**4**: Blackbelly rosefish, Witch flounder	Mid-trophic level species; bathydemersal, found in very deep waters.

**Table 2:** Description of core species (i.e., identified in the cluster by both k-means and hierarchical clustering and in at least three out of four periods) found in each species assemblage defined for the northern and southern regions of the spring bottom trawl survey.

**North: Gulf of Maine/Scotian Shelf**	
**Assemblage**	**Description**
**1**: Atlantic herring, Cunner, Little skate, Longhorn sculpin, Windowpane flounder, Winter flounder, Yellowtail flounder	Mid-trophic level species; mainly demersal; primarily in shallower, coastal inshore waters/protected bays and estuaries; some spawn in estuaries and rivers.
**2**: Alewife, American plaice, Fourspot flounder, Haddock, Wolffish	Mix of mid- and higher trophic level species; mix of pelagic and demersal species; found more frequently in coastal inshore waters.
**3**: Acadian redfish, Cusk, Monkfish, Red hake, Silver hake, Witch flounder	Mainly higher trophic level species; generally demersal, mid to deep waters and occasionally/soft bottom.
**4**: Blackbelly rosefish, White hake	Mid-trophic level species; bathydemersal, found in very deep waters.
**South: Mid-Atlantic Bight/Georges Bank**	
**1**: Atlantic croaker, Blueback herring, Bluefish, Clearnose skate, Little skate, Northern kingfish, Pin fish, Spot, Striped bass, Tautog, Weakfish, Windowpane flounder, Winter flounder, Winter skate	Mix of mid- and higher trophic levels; mainly demersal and reef-associated; strongly tied to coastal inshore waters, bays, estuaries, etc. and warm waters.
**2**: American lobster, American plaice, Barndoor skate, Black sea bass, Butterfish, Fourspot flounder, Gulfstream flounder, Longfin squid, Red hake, Spiny dogfish, Thorney skate	Mix of mid- and higher trophic level species; mainly demersal/benthic; Generally in coastal waters, or have the ability to migrate between deeper and shallower depths.
**3**: Rosette skate, White hake	Mid- to high trophic levels; Found mostly around shelf edge over soft bottoms.
**4**: Blackbelly rosefish, Witch flounder	Mid-trophic level species; bathydemersal, found in very deep waters.

The exact species in each assemblage are generally similar within a given region, but differ slightly between seasons. Depth is the strongest variable delineating the assemblages and shows a consistent pattern between seasons and regions (Fig 2). Therefore, the gradation in depth was used to differentiate between the assemblages, and assemblages are numbered 1 to 4 to reflect groups of species associated with progressively deeper waters. We use these numbers in combination with the letter ‘N’ or ‘S’ to denote whether a particular assemblage is associated with the northern or southern NES, respectively. Notably, species in the Gulf of Maine (northern NES) assemblages (denoted in the text as 1N-4N) differ from the Mid-Atlantic Bight/Georges Bank (southern NES) assemblages (denoted in the text as 1S-4S), i.e., species in 1N do not correspond to those in 1S, but species in assemblages 1 to 4 in both regions follow the same gradation of shallower to deeper depths. Differences in bottom temperature also characterize the assemblages, although the patterns differ somewhat between seasons (Fig 2). In the fall across both regions, species in assemblage 1N and 1S are found in areas with warmer bottom temperatures, while assemblages 2, 3, and 4 are found in areas with progressively cooler bottom temperatures. In the spring in the southern NES, there is little difference between the bottom temperatures occupied by the species in each of the assemblages, although species in assemblage 1S occupy a wider range of bottom temperatures and some are associated with much warmer temperatures than the other assemblages. In the spring assemblages for the northern NES, assemblage 1N is associated with the coolest bottom temperatures and assemblages 2N, 3N, and 4N are associated with progressively warmer bottom temperatures, a pattern that is opposite to what is observed for the fall. Differences in surface temperature have the least effect on delineating the assemblages in either season or region, although species in assemblage 1 seem to be associated with somewhat warmer surface temperatures (Fig 2). These depth and thermal patterns are consistent regionally and seasonally across the four time periods (S4 Fig).

In general, assemblages 1N and 1S consist of shallow water demersal species generally associated with warmer bottom and surface temperatures, e.g., species found in coastal and/or protected waters such as bays and estuaries (Tables 1 and 2). Additionally, in the southern NES, assemblage 1S consists of some reef-based species (Tables 1 and 2). Assemblages 2N and 2S consist of mid-shallow water, mid- and higher trophic levels species, which are generally coastal, but with the ability to move between shallower and deeper waters (Tables 1 and 2). Assemblages 3N and 3S consist of mid-deep water, demersal, higher tropic level species, found over soft bottom habitat (Tables 1 and 2). This group inhabits deeper waters in the northern NES and is found along the shelf edge in the southern NES. Assemblages 4N and 4S are characterized by deep-water species associated with colder bottom temperatures (Tables 1 and 2).

**Fig 3:**
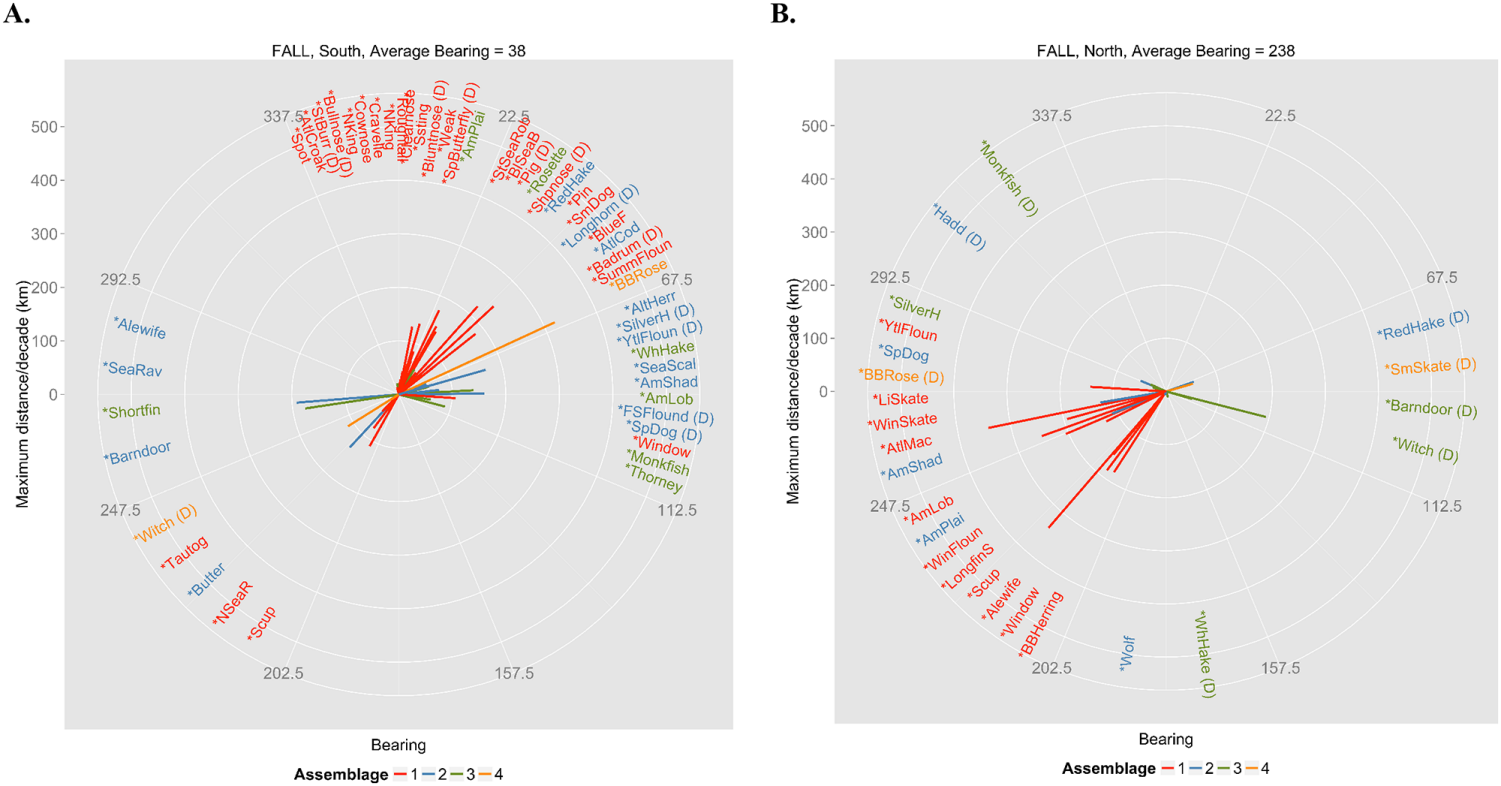
Fall compass plot. Bearing (0–360 degrees) and distance (km) between the centers of biomass in the first (1968–1978) and fourth (2001–2012) periods for each core species for (A) the Mid-Atlantic Bight and Georges Bank (southern NES) and (B) the Gulf of Maine (northern NES) sampled during the NEFSC fall bottom trawl surveys. Full species names corresponding to abbreviations are in S1 Table. The presence of a ‘(D)’ after an abbreviation refers to a species that has a significant deepening trend over the entire time series determined by linear regression.

**Fig 4:**
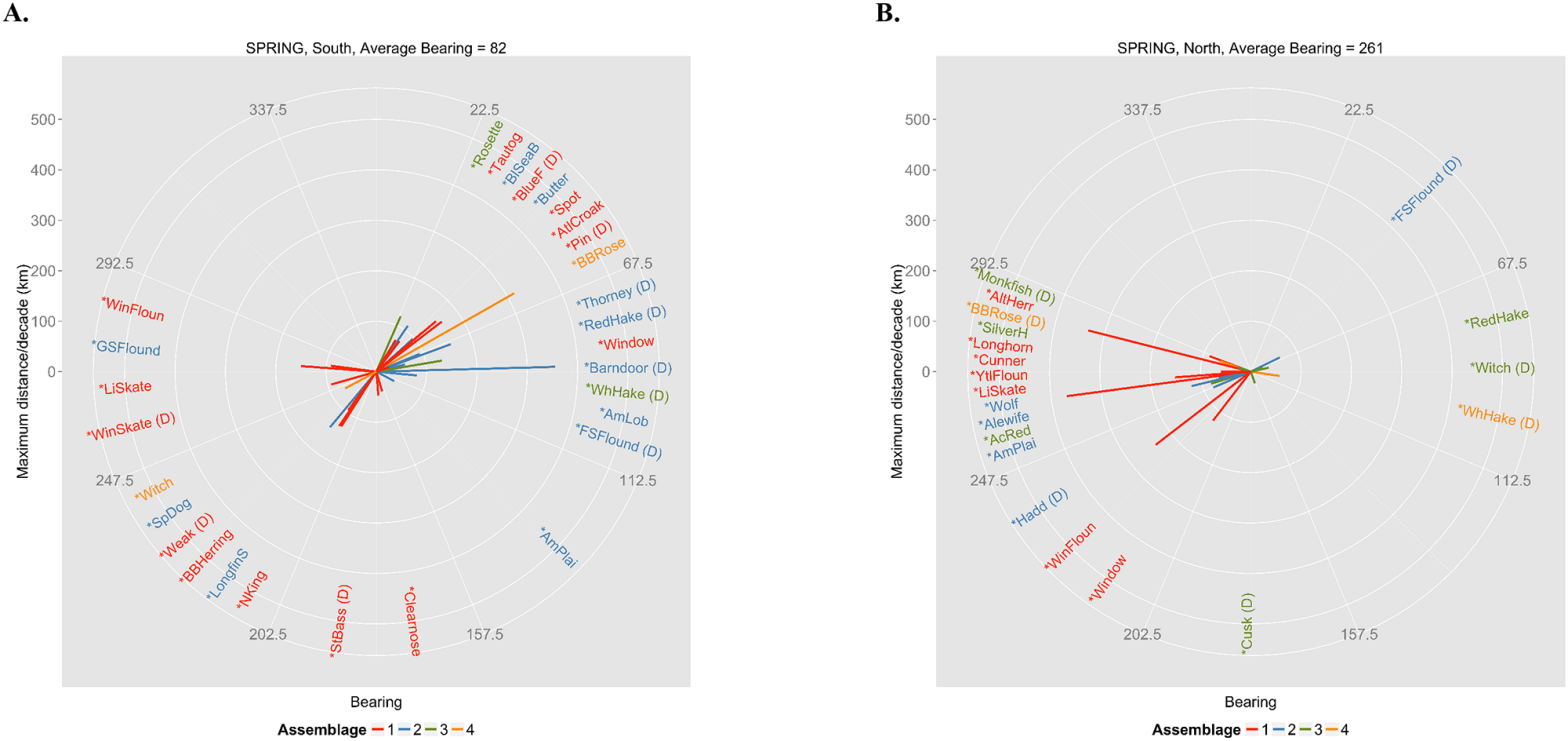
Spring compass plot. Bearing (0–360 degrees) and distance (km) between the centers of biomass in the first (1968–1978) and fourth (2001–2012) periods for each core species for (A) the Mid-Atlantic Bight and Georges Bank (southern NES) and (B) the Gulf of Maine and U.S. Scotian Shelf (northern NES) sampled during spring bottom trawl surveys. Full species names corresponding to abbreviations can be found in S1 Table. The presence of a ‘(D)’ after an abbreviation refers to a species that has a significant deepening trend over the entire time series.

Between 1960 and 2014, surface and bottom temperatures across the NES warmed by approximately 2°C [44]. Although directions of movement vary to some degree between and within regions (Figs 3 and 4), shifts are generally toward the northeast and east along the Mid-Atlantic Bight/Georges Bank (southern NES; fall: Fig 3A average bearing 38°; spring: Fig 4A, average bearing 82°) and west-southwest and west in the Gulf of Maine (northern NES; fall: Fig 3B, average bearing 238°; spring: Fig 4B, average bearing 261°). However, the direction of shift varies between the assemblages.

**Fig 5:**
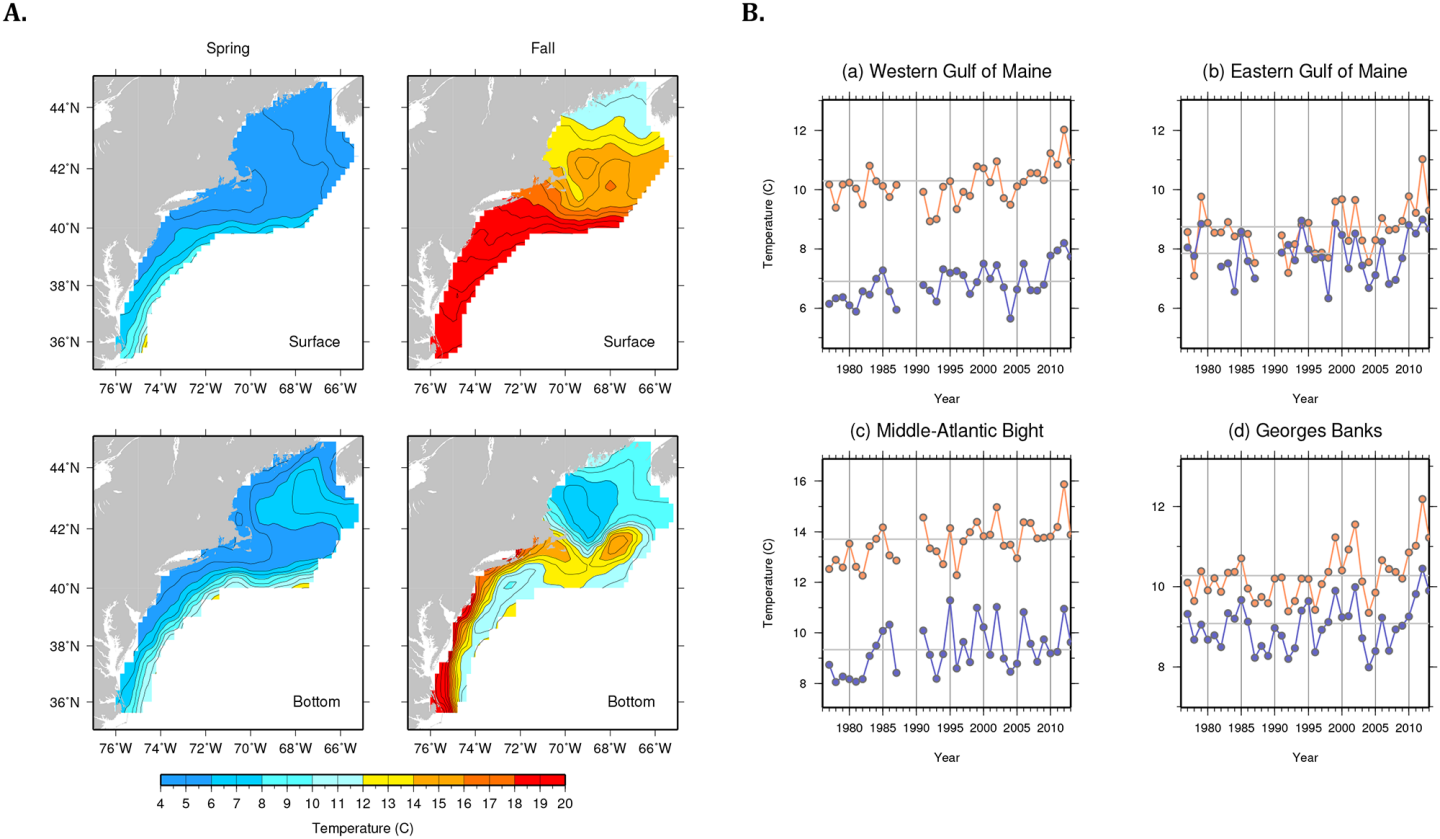
Bottom and surface temperature on the U.S. Northeast Shelf. (A) Interpolated, average (1977–2013) surface and bottom temperatures on the northeast shelf from the NEFSC spring and fall bottom trawl surveys. (B) Regional time series of bottom temperature (blue) and surface temperature (orange) computed as area-weighted means of all survey points within a given region. The horizontal lines in each panel represent the average over the reference period (1977–2013).

In the southern NES in the fall, species in assemblage 1 are predominantly shifting to the north and northeast, while species in assemblages 2 and 3 are shifting east with some species in assemblages 1, 2, and 3 exhibiting deepening trends (Fig 3A). Species in assemblage 4 show more variability: blackbelly rosefish is shifting northeast, while witch flounder is shifting southwest, but also deepening (Fig 3A). Conversely, in the southern NES in the spring, the shifts among species in assemblage 1 exhibit much more variability in terms of direction of shift (Fig 4A). The most significant distances shifted for species in assemblages 2 and 3 are to the east and northeast, with many of those species exhibiting deepening trends (Fig 4A). In the northern NES across both seasons, species in assemblage 1 are shifting west-southwest (Figs 3B and 4B). With the exception of red hake in the spring, species in assemblages 2, 3, and 4 that are shifting in directions other than west and southwest are exhibiting a deepening trend (Figs 3B and 4B). Within the shallower assemblages (i.e., 1 and 2) latitudinal shifts are more clustered in the dominant direction of movement according to Rayleigh’s z test (1N: z-statistic: 0.91, p-value < 0.0001; 2N: z-statistic: 0.47, p-value = 0.013; 1S: z-statistic: 0.19, p-value = 0.040; 2S: z-statistic: 0.30, p-value = 0.018). However, there are no statistically significant modes of shift direction within the deeper water assemblages, possibly because many of these species are shifting to a greater extent along the vertical axis of the water column as signified by significantly deepening trends over the time series (i.e., species denoted by a “(D)” in Figs 3 and 4). In the absence of strong physiographic constraints in the Mid-Atlantic Bight/Georges Bank region, shifts in distribution largely follow the prevailing shift in temperature isotherms toward the northeast. Conversely, west-southwester shifts in the Gulf of Maine may reflect cooler bottom temperatures in the southwestern Gulf of Maine (Fig 5A) over the time period (Fig 5B and S5 Fig).

**Fig 6:**
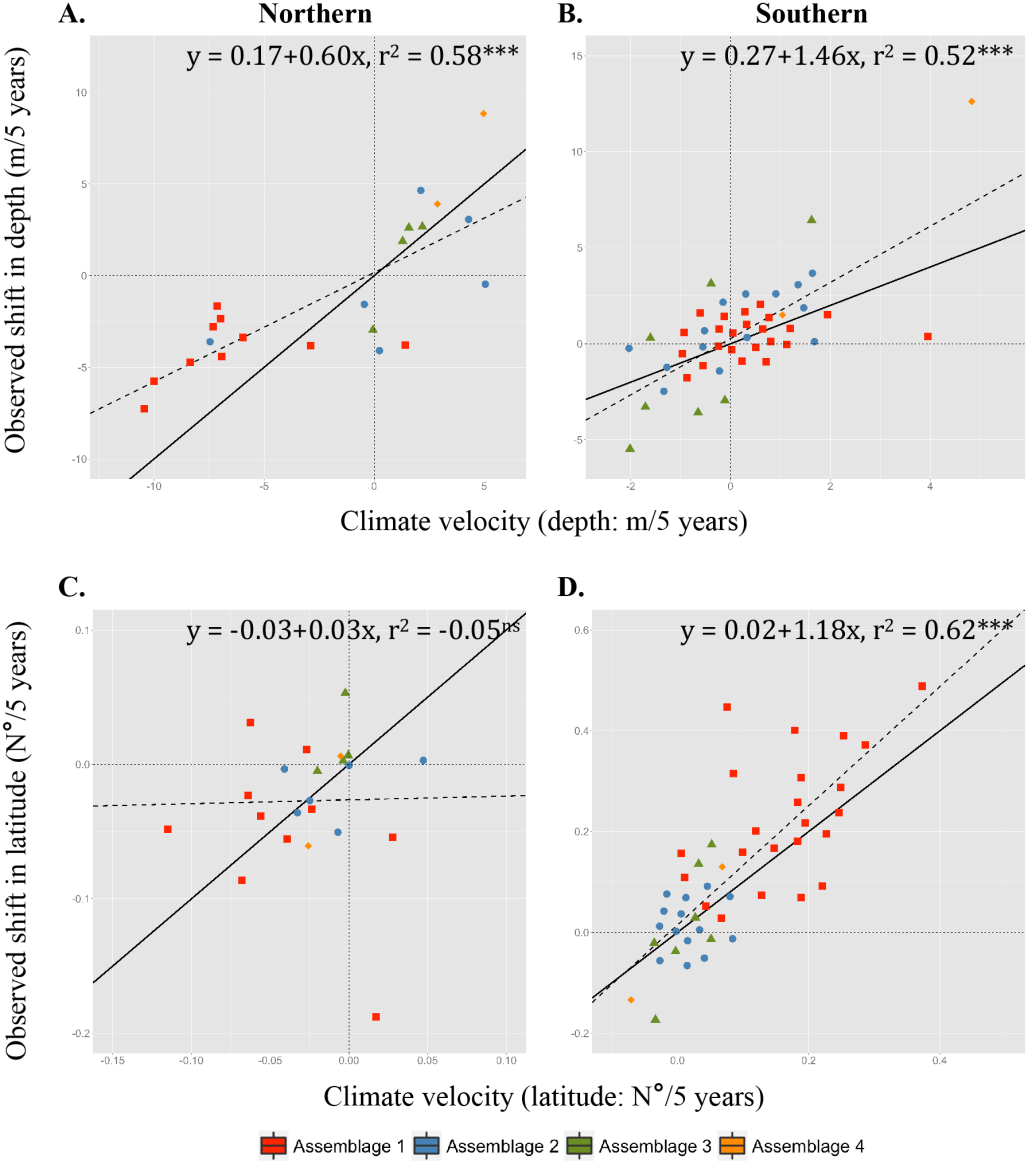
Fall climate velocities. Slopes of observed versus predicted changes in depth (A, B) and latitude (C, D) for the northern NES: Gulf of Maine (A, C), and southern NES: Mid-Atlantic Bight/Georges Bank (B, D) sampled during the NEFSC fall bottom trawl surveys. Colors correspond to clusters (red: cluster 1N or 1S; blue: cluster 2N or 2S; green: cluster 3N or 3S; yellow: cluster 4N or 4S). Significance is indicated by ‘ns’: not significant; ‘*’: p < 0.05; ‘**’: p < 0.01; ‘***’: p < 0.001. The solid black line is the 1:1 relationship and the dashed black line corresponds to the linear model fit and provides a reference point for whether the clusters are moving faster or slower relative to climate velocity with respect to latitude and depth.

**Fig 7:**
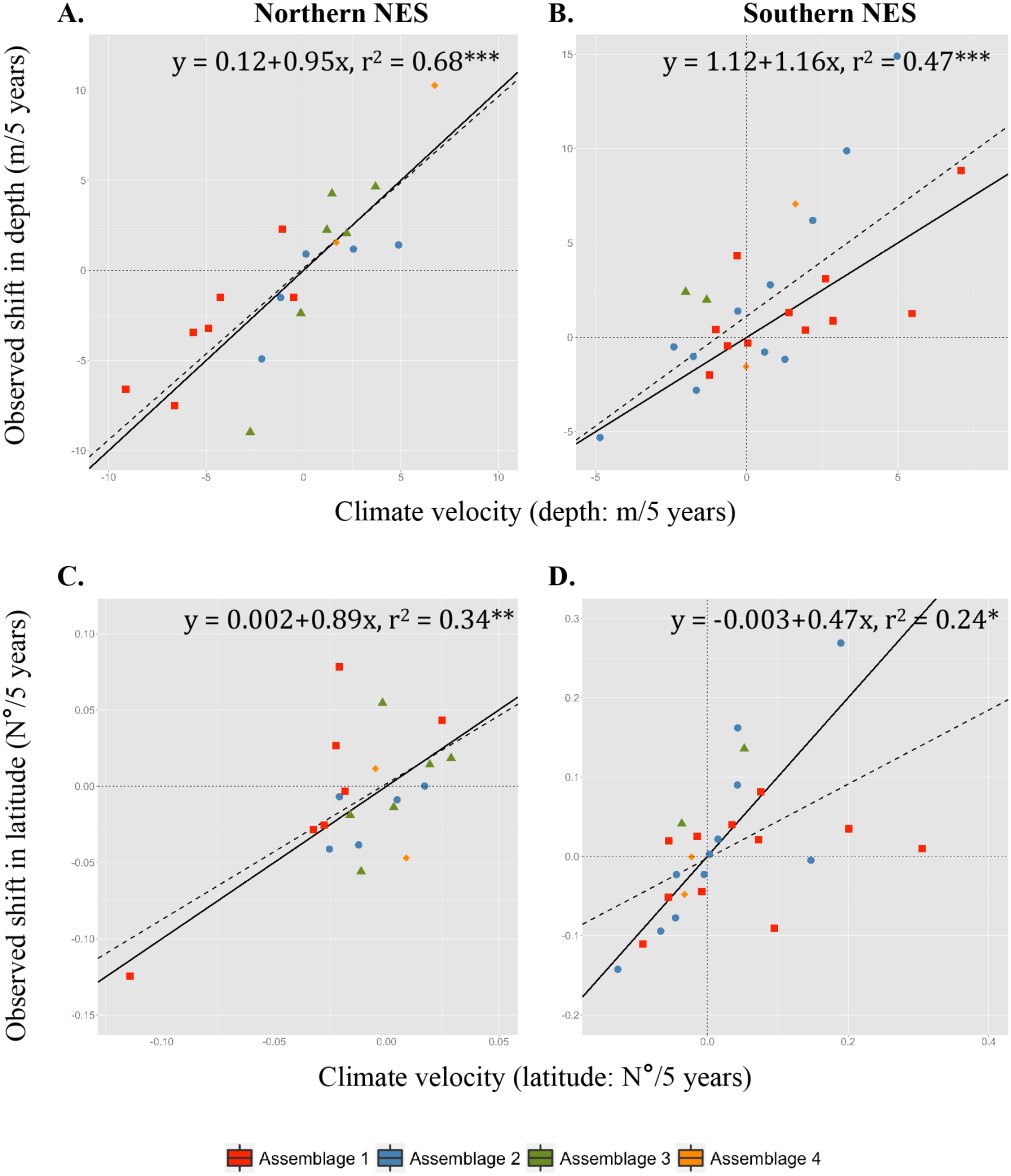
Spring climate velocities. Slopes of observed versus predicted changes in depth (A, B) and latitude (C, D) for the Gulf of Maine (northern NES; A, C) and Mid-Atlantic Bight/Georges Bank (southern NES; B, D) sampled during the spring bottom trawl surveys. Colors correspond to the species clusters (red: cluster 1S; blue: cluster 2S; green: cluster 3S; yellow: cluster 4S). Significance is indicated by ‘ns’: not significant; ‘*’: p < 0.05; ‘**’: p < 0.01; ‘***’: p < 0.001. Solid black line is the 1:1 relationship and dashed black line corresponds to the linear model fit and provides a reference point for whether the clusters are moving faster or slower relative to climate velocity with respect to latitude and depth.

In the Gulf of Maine where physiographic constraints are strong, depth-related shifts are stronger than latitudinal shifts, with observable differences between species assemblages. The relationship with depth is strong and significant in the fall (Fig 6A; r^2^ = 0.58, p < 0.001) and a similar relationship is found in the spring (Fig 7A; r^2^ = 0.68, p < 0.001). Generally, most species in the deeper-water assemblages (N3 and N4) are deepening (Figs 6A and 7A), while all of the species in the shallow-water assemblage (N1) are getting shallower (Figs 6A and 7A). Species in assemblage N2 showed the greatest variability in terms of shifts in depth in both seasons (Figs 6A and 7A). While species in shallow-water assemblages 1N are moving west-southwest (Figs 3B and 4B), possibly tracking cooler bottom temperatures in the southwestern Gulf of Maine (S5 Fig), overall there is no significant relationship in terms of latitudinal climate velocities in the fall (Fig 6C; r^2^ = -0.05, p > 0.05), and only a weakly significant relationship in the spring (Fig 7C; r^2^ = 0.34, p < 0.01). These patterns are supported by the fact that there are distinct differences between depth-based climate velocities in the northern NES between the assemblages in both seasons (fall: *chi*-squared = 11.57, p = 0.009; spring: *chi*-squared = 10.87, p = 0.012), but no differences between assemblages in terms of latitudinal shifts in either season (fall: *chi*-squared = 4.73, p = 0.193; spring: *chi*-squared = 6.56, p = 0.087).

In the Mid-Atlantic Bight/Georges Bank region, species display significant correlations between shifts in depth and species-specific climate velocities in the fall (Fig 6B; r^2^ = 0.34, p < 0.001) and in the spring (Fig 7B; r^2^ = 0.47, p < 0.001). Comparison of linear model fits with the 1:1 line indicates most species are deepening slightly faster than what climate velocities would suggest in both seasons. Along the southern NES region, where depths are more homogeneous and bottom temperatures are horizontally aligned along a southwest-northeast gradient, shallow-water species assemblages are moving northeastward, strongly tracking climate velocity (Fig 6D; r^2^ = 0.62, p < 0.001). In the spring, there is a weakly significant relationship with latitude (Fig 7D; r^2^ = 0.24, p < 0.05), but most species are shifting slower than what climate velocity would suggest with little pattern by assemblage. These seasonal differences may be attributed in part to the fact that on the NES, winter surface temperatures have shown much lower variability than surface temperatures in the summer, which have warmed substantially [44]. There are significant differences between assemblages in terms of latitudinal-based climate velocities in the fall, but not the spring (fall: *chi*-squared = 27.01, p < 0.0001; spring: *chi*-squared = 1.04, p = 0.791), and no differences between assemblages in terms of depth-related shifts in either season (fall: *chi*-squared = 7.58, p = 0.055; spring: *chi*-squared = 3.98, p = 0.263).

**Fig 8:**
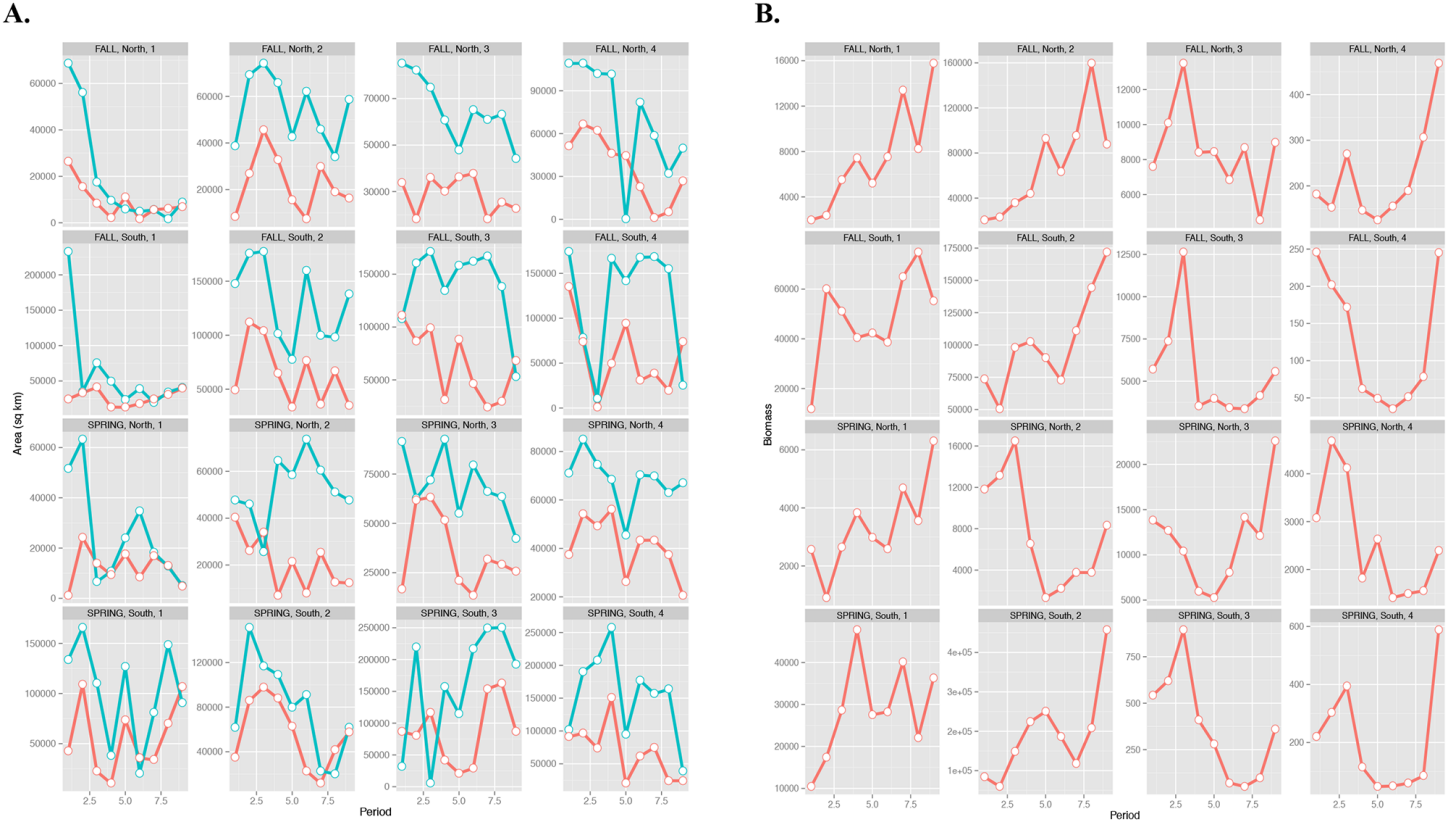
Assemblage spatial extent, thermal area, and biomass. (A) Trends in five-year averages of assemblage spatial extent (blue lines) and predicted thermal habitat area (red lines). Area is defined by the kernel densities of the assemblage biomass with values greater than one standard deviation above the mean for the assemblages defined in each season and region. (B) Comparative trends five-year averages of summed biomass for each assemblage.

To examine fluctuations in the spatial extent of the assemblages versus changes in the extent of thermal habitat and assemblage biomass, a linear mixed effects model is used to determine whether either the biomass of a given assemblage or the area of thermal habitat is a better predictor of the spatial extent of each assemblage. Thermal habitat is strongly significant and positively related to assemblage area (t = 3.72, *p* = 0.003) while the biomass of the assemblage is not significant in the model (*p* = 0.882), indicating that climate rather than abundance is having a greater effect on the assemblage extent. Temporal trends in the spatial extent of the assemblages often counter biomass trends, but mirror trends in the extent of the thermal habitat area (Fig 8), a result that counters MacCall’s basin hypothesis [29] and species-level studies that have shown a positive relationship between range size and abundance [30, 45].

## Discussion

The patterns described here illustrate consistencies in climate-related changes between groups of species that share similar bathy-thermal characteristics, especially among species associated with shallower waters. Within the NES, the role of physiographic constraints and local oceanography reveal important sub-regional differences in the direction of shift, with species in the physiographically constrained Gulf of Maine shifting generally west-southwest and species along the southern NES shifting generally to the northeast. In both regions, local climate variability is likely a strong driver, and for many species, rates of latitudinal and depth-related shifts tracked predicted climate velocities.

The shifts observed here might be subject to bias due to the fact that we are limited to observations within a set sampling domain. This is an issue faced in all sampling programs in marine systems previously employed for this purpose, but not previously considered by other studies using data from a specific sampled area to explore species shifts. The application of truncated regression models (see S1 Appendix) in this region serves to illustrate whether the observed southern latitudinal shifts might be stronger given the possibility. The results from this analysis illustrate that this does not seem to be the case (S7 Fig).

West-southwesterly shifts in the Gulf of Maine may reflect cooler bottom temperatures in the southwest Gulf of Maine that have been observed over the time period (Figs 5 and 6 and S5 Fig). Cooler bottom temperatures are a function of basin geometry and seasonal cycles of temperature and salinity in the western Gulf of Maine, which support deeper mixing of cooler surface waters in the winter and stronger stratification in the summer [46, 47]. Prevailing winter winds blow off the continental landmass, preferentially extracting heat from the surface waters in the western Gulf of Maine where air-sea temperature contrast is largest. This occurs seasonally when surface salinities in the western Gulf of Maine are maximized relative to the eastern basin, enabling deeper mixing of winter-cooled waters [47].

Species associated with shallower waters (i.e., assemblages 1 and 2) show more consistency in terms of their direction of shift. This result may indicate that species associated with deeper waters may have more temperature refuges available and/or the ability to shift to deeper waters if they offer suitable habitat. Significant deepening trends are observed more frequently among species associated with deeper waters, which suggests that this type of adaptation may be more available to deeper water species. In terms of rates of shift, species in assemblage 1 in the southern NES in the fall are shifting strongly northward and largely maintaining the same depths, with some species shifting as fast as 0.1°N per year. This pattern was not clear in the spring, and this is probably because enhanced warming has occurred in the late summer and early fall since the 1980s [12]. Therefore, we expected that the fall survey would be able to pick up signals in climate velocity and species shifts more clearly than the spring survey. In the northern NES across both seasons, species in assemblage 1 are shifting to shallower waters and in a southerly direction, whereas species in the deeper assemblages tend to illustrate shifts deeper. The strong shift to deeper waters exhibited by many of the deeper water species may indicate that basins like Georges in the western Gulf of Maine, and Wilkenson and Jordan basins in the eastern Gulf of Maine, offer deep-water refuges for species that are able to shift to deeper waters. The shift of shallow-water species to shallower waters may be due to competition from species entering the Gulf of Maine or shifting from other parts of the Gulf of Maine has been increasing as these newcomers take advantage of cooler bottom temperatures in the southwestern portion of the Gulf of Maine. If the shallower water species are unable to move to deeper waters either because of physiological constraints or species interactions then they may be pushed into shallower waters.

As species within the assemblages expand or contract and shift into new regions, they may be subjected to a respective reduction or enhancement in levels of species interactions, resulting in further pressures on stocks as they experience higher or new sources of competition or reduced levels of prey. The correlation of regional assemblage range sizes with thermal habitat area suggests that climate is a key driver of change in this system. Sub-regional differences in responses to climate change illustrate that information on the rate and direction of community- and species-level shifts at relevant spatial scales will be critical for fisheries management, which is typically conducted at sub-regional scales consistent with stock structures and jurisdictional boundaries.

The fact that we did not see a significant and positive relationship between the spatial extent of the assemblages and biomass is contrary to MacCall’s basin hypothesis [29], which hypothesizes that the geographic area that a species inhabits is directly related to its population size, and species-level studies that have shown a positive relationship between range size and abundance [30, 45]. Our results suggest that thermal areas that are ideal for particular assemblages are decreasing over time, corresponding to decreases in the spatial extent of some assemblages (Fig 8). Previous studies have suggested that, in areas where thermal habitat area is decreasing, inter- and intra-specific interactions may be significantly strengthened as species within assemblages are compressed into smaller areas of suitable habitat [26–28]. For example, increased competition as a result of rising temperatures have been shown to be an issue in montane habitats where less cold-tolerant plant species may shift into smaller regions of higher altitude and compete with species already present in these areas [48]. Strong climate velocity responses at the assemblage level, such as those exhibited for the shallow, warmer bottom water assemblages (1S) in the southern NES, in combination with an increase in density (i.e., greater abundance and a contraction in spatial extent), may result in heightened species interactions. For example, such a scenario may result in changes in the interaction rates between predators and prey [49, 50]. The use of trawl survey data in this study allows for the simultaneous evaluation of species at different trophic levels (e.g., sea scallops at trophic level 2 and Atlantic cod at trophic level 4.4, S1 Table), a feature that is critical when considering species interactions.

Within the assemblages, variability in the rates of shift of individual species could indicate some decoupling of trophic interactions [48]. Extending this work to include explorations of more trophic levels (i.e., zooplankton and large pelagic predators) will be necessary to illicit more nuanced trophic dynamics. For example, studies have shown that individual species that modify their behavior to avoid stressful conditions or exposure, or that must increase foraging rates and extent to meet metabolic needs, will alter encounter rates among species [27]. Therefore, gaining an understanding of the relative rates of shift of both predators and prey may reveal whether there are mismatches that could have negative consequences for the feeding success of predators or, alternatively, reduced predation rates conferring better survival for prey [26]. Additionally, shifts in distribution of one life history stage may affect the connectivity between other stages and have significant impacts on processes such as spawning and recruitment [51]. In the U.S. NES, larval fish distributions changes have been relatively consistent with expectations from a changing climate [51]. Another important consideration is whether species that shift more quickly relative to climate velocity have greater dispersal ability [52]. If this is the case, then species that are able to move with or faster than shifts in isotherms may indicate species that are better able to adapt and shift to find suitable environments or species that are more sensitive to temperature changes. Visualizing patterns in rates of shift across groups of species can highlight whether particular habitat preferences confer a competitive advantage for climate adaptation.

Fishing pressure could be considered to be another source of ‘predation’ on many marine species, and it is possible that the shifts observed here are driven by fishing rather than climate. One way to attempt to understand the possible influence of fishing pressure on fish distributions is to examine the spatial distribution of fishing effort over the time series to determine whether areas of higher effort correspond to areas where we see a decline in abundance. Information on the spatial distribution of fishing effort is not available prior to the 1990s. However, the spatial distribution of fishing effort over the last two decades has declined along the southern Mid-Atlantic Bight and eastern Gulf of Maine, regions that are counter to the dominant directions of sub-regional species shifts (S6 Fig). This suggests that fishing pressure is less of a driver than climate, as one would expect that fish populations would be more depleted in areas where fishing is heavier if fishing pressure is the stronger driver [46, 47]. Interestingly, fishing effort in the western Gulf of Maine has increased over the deep Wilkinson Basin (S6 Fig). The climate velocities in the northern NES illustrate the importance of depth-related shifts in the Gulf of Maine and may indicate that species are strongly tracking cooler temperatures in these deep basins despite the concentration of fishing effort in these areas. However, the contraction in range extent illustrated for some of the assemblages described here may eventually result in increased vulnerability to fishing activity, e.g., the shift of Atlantic cod (*Gadus morhua*) distribution into northern Gulf of Maine waters, which has resulted in a decline in abundance [5, 25]. Along the Mid-Atlantic Bight, shifting distributions of traditionally harvested species will alter patterns of availability to local fishing communities, imposing economic impacts as a result of lost access to stocks managed with species-specific quotas, and rising fuel and travel costs [53].

The analyses presented here illustrate that species-level responses to climate change are generalizable among taxonomic groups and across regions. Species assemblages defined by similar bathy-thermal characteristics can be used to illustrate cohesive climate change responses at the community level. Additionally, by explicitly accounting for sub-regional physiographic constraints and oceanography, it is possible to evaluate differences in assemblage-level distributional shifts in terms of depth and latitude. Changes in assemblage ranges that correspond to changes to thermal area availability have important consequences for species interactions and the level of fishing effort concentrated on fish stocks. Therefore, the ability to distinguish regional climate responses at the community-level provides important information for ecosystem-based fisheries management.

## Supporting Information

S1 Appendix Methodology for observed species shifts versus climate velocity using truncated regressions. (DOCX)

S1 Fig Annual NEFSC bottom trawl survey coverage for the spring. Black dots represent a sampled site in each year.
(PDF)

S2 Fig Annual NEFSC bottom trawl survey coverage for the fall. Black dots represent a sampled site in each year.
(PDF)

S3 Fig ‘Hotspots’ of cluster locations in each region. Red signifies clusters 1N and 1S, blue signifies clusters 2N and 2S, green signifies clusters 3N and 3S, and yellow signifies clusters 4N and 4S. The Gulf of Maine (northern NES) is shown in the top panels, and the Mid-Atlantic Bight/Georges Bank (southern NES) is shown in the bottom panels. Hotspots are based on kernel density values greater than one standard deviation above the mean. Black lines correspond to the 100 and 200m isobaths.
(PDF)

S4 Fig Characterization of the species assemblages derived from the NEFSC bottom trawl survey over four periods (1968–1978, 1979–1989, 1990–2000, and 2001–2012) in three-dimensional space. Assemblages are defined by surface temperature (x-axis), bottom temperature (y-axis), and depth (z-axis) in the Fall (A, B) and Spring (C, D) in the northern (A, C) and southern (B, D) NES.
(PDF)

S5 Fig Description of the bottom temperature fields on the NES. Comparison of average bottom temperature fields in the fall on the U.S. Northeast Shelf for an early part of the time series (1977–1987) and a later part of the time series 2000–2010. The bottom panel shows the difference field (late minus early).
(PDF)

S6 Fig Percent change in effort by the U.S. otter trawl fishery from 1995 through 2015. Blue (red) colors indicate a decrease (increase) in fishing effort between respective fishing periods. The EEZ is illustrated as a red line and the 200 m isobaths as a light grey line. Black lines illustrate the closed area boundaries. In general fishing effort has decreased in many area of the U.S. Northeast Shelf over the past two decades.
(PDF)

S7 Fig Latitudinal climate velocities based on a truncated regression. Slopes of observed versus predicted changes in latitude from truncated regressions for the Gulf of Maine (northern NES; a, b) and Mid-Atlantic Bight/Georges Bank (southern NES; c, d) northeast U.S. shelf sampled during spring (a, c) and fall (b, d) bottom trawl surveys. Colors correspond to clusters (red: cluster 1N or 1S; blue: cluster 2N or 2S; green: cluster 3N or 3S; yellow: cluster 4N or 4S). Significance is indicated by ‘ns’: not significant; ‘*’: p < 0.05; ‘**’: p < 0.01; ‘***’: p < 0.001. Significance is indicated by ‘ns’: not significant; ‘*’: p < 0.05; ‘**’: p < 0.01; ‘***’: p < 0.001. Solid black line is the 1:1 relationship and dashed black line corresponds to the linear model fit and provides a reference point for whether the assemblages are moving faster or slower relative to climate velocity with respect to latitude and depth.
(PDF)

S1 Table Description of the key species in the species clusters defined from the bottom trawl survey. The presence of a ‘1’, ‘2’, ‘3’, or ‘4’ in the columns ‘Spring, South’, ‘Spring, North’, ‘Fall, South’, or ‘Fall, North’ indicates membership of a core species to a particular cluster. An ‘xx’ indicates that the species is present in a given region, but was not identified as a core species in any cluster and a blank entry indicates that the species is not present in either season in a given region.
(DOCX)
