# Supplementary material for: The Effects of Sub-Regional Climate Velocity on the Distribution and Spatial Extent of Marine Species Assemblages
Source: PLoS One. 2016 Feb 22;11(2):e0149220. doi: 10.1371/journal.pone.0149220 (PMC4762943; doi:10.1371/journal.pone.0149220)
Supplement: S1 Appendix — (DOCX) [file pone.0149220.s001.docx]

**S1 Appendix**

*Observed species shifts versus climate velocity using truncated regressions*

In some situations, data on a particular variable may not exist or be available above or below a certain limit, i.e., the data are truncated. In these cases, a regression using this variable as a response may be biased and special estimators of the regression coefficient may be needed. This is the case in this study for latitudes north or south of the survey region for which observations do not exist or in regions where there is a geographic constraint (i.e., a land mass) to further movement. This situation may induce a bias if species would actually move further north or south than predicted by a regular linear regression given the ability to do so or the ability to track this movement. Here, in order to test the effect of truncation bias on species shifting southward in the Gulf of Maine/Scotian region or northward in the Mid-Atlantic Bight/Georges Bank region, we replace the ordinary linear regression of biomass-weighted mean latitude and prediction-weighted mean latitude used, respectively, for determining the observed species shift and taxon-specific climate velocity with a semi-parametric truncated linear regression model in R with package truncSP [[1](#_ENREF_1)]. For the Mid-Atlantic Bight/Georges Bank region, we define a right truncation (i.e., north truncation) at 42°N to explore whether the northward response would be dampened or heightened. For the Gulf of Maine, we define a left truncation (i.e., south truncation) at 41°N to explore whether the southward response would be dampened or heightened. Figure S10 illustrates the relationships between the slopes determined from the truncated regressions for spring and fall for both regions.

In the Gulf of Maine, in the spring, there is a weakly significant relationship with latitude with the ordinary linear regression (Fig 7C: R^2^ = 0.34, p <0.01), but no significant relationship with the left truncated regression (Fig. S7A). The result for the fall remains nearly the same with no significant relationship with either the ordinary linear regression or the left truncated regression (Fig 6C; Fig. S7B). In the Mid-Atlantic Bight/Georges Bank region, the patterns remain identical for spring and fall, i.e., shallow-water species move northward at the greatest rates, strongly tracking climate velocity, when we apply an ordinary linear regression or a right truncated regression. In the spring the relationship is less significant with the ordinary linear regression (Fig. 7D: R^2^ = 0.24, p <0.01) and this relationship is slightly stronger with the right truncated regression (Fig S7C: R^2^ = 0.32, p <0.01). For fall the relationship with the ordinary linear regression is strongly significant (Fig. 6D: R^2^ = 0.62, p <0.001) and the relationship with the right truncated regression is significant, but slightly weaker (Fig. S7D: R^2^ = 0.45, p <0.001).

The application of truncated regression models in the NES serves to illustrate whether the observed southern latitudinal shifts might be stronger given the possibility for a species to move further north or south or the possibility to sample further north or south. The results from this analysis illustrate that this does not seem to be the case.
