# Supplementary figures and images for: The Effects of Sub-Regional Climate Velocity on the Distribution and Spatial Extent of Marine Species Assemblages
Source: PLoS One. 2016 Feb 22;11(2):e0149220. doi: 10.1371/journal.pone.0149220 (PMC4762943; doi:10.1371/journal.pone.0149220)

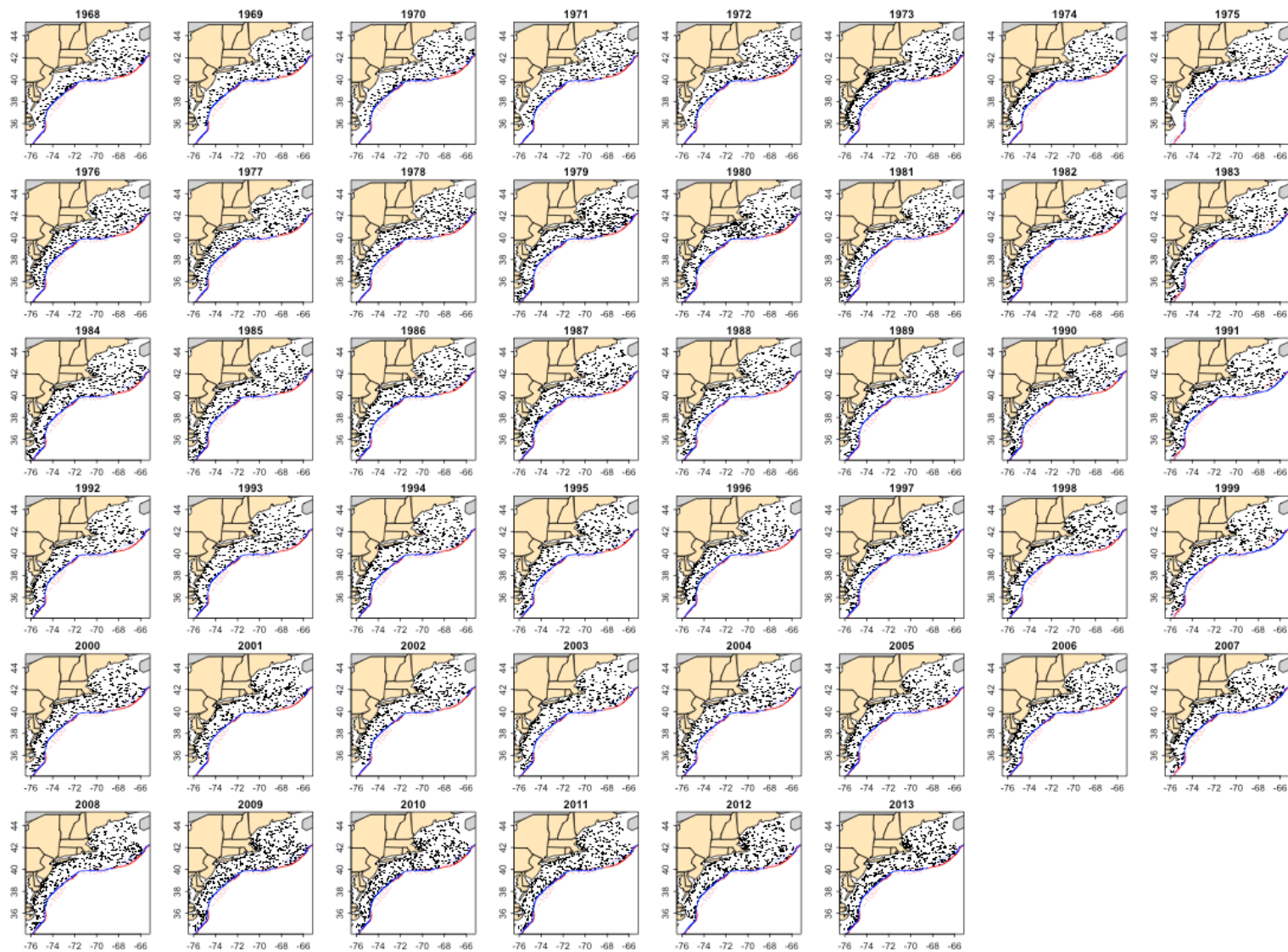

Supplement: S1 Fig — Black dots represent a sampled site in each year. (PDF) [file pone.0149220.s002.pdf]

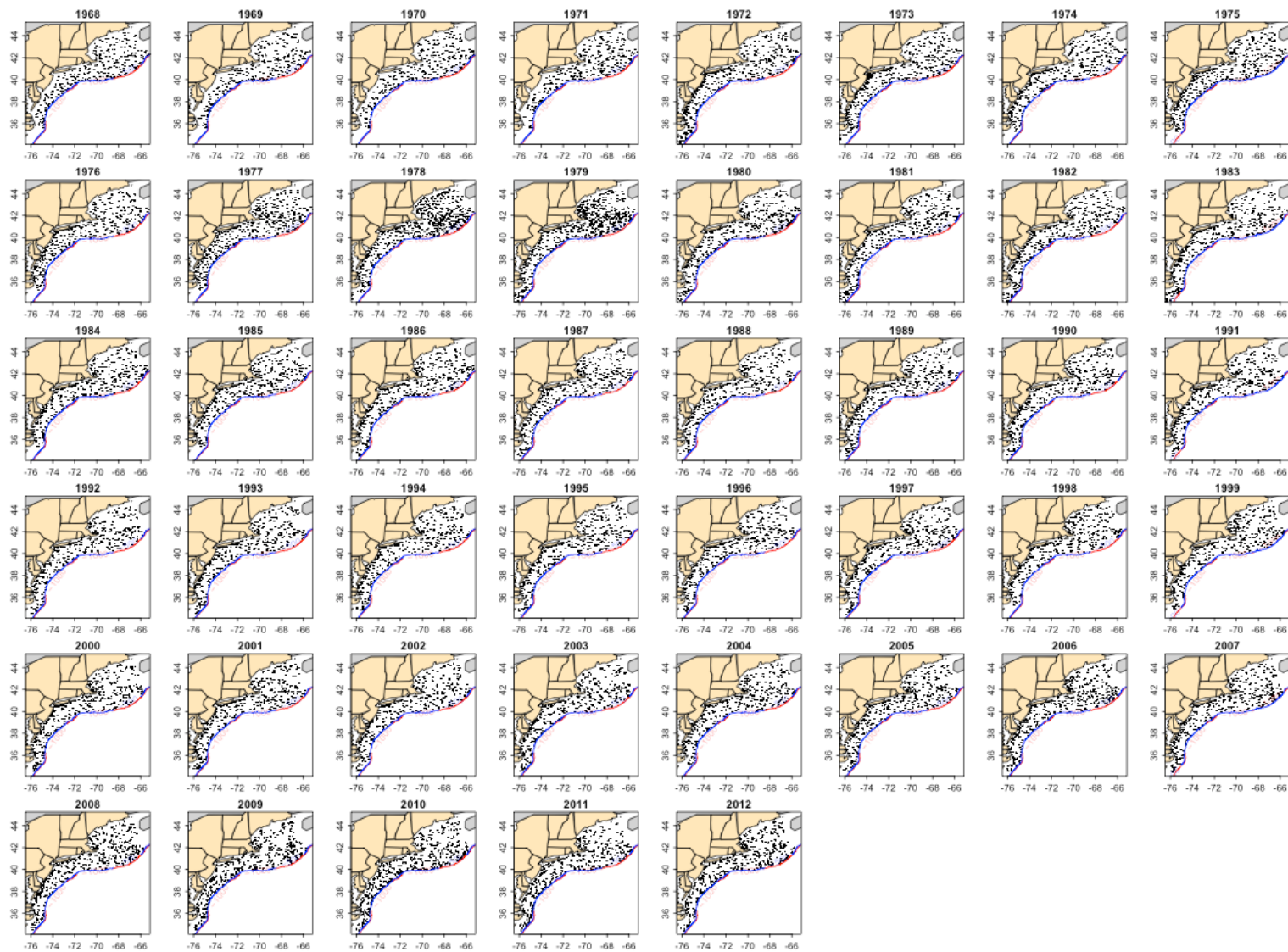

Supplement: S2 Fig — Black dots represent a sampled site in each year. (PDF) [file pone.0149220.s003.pdf]

Spring

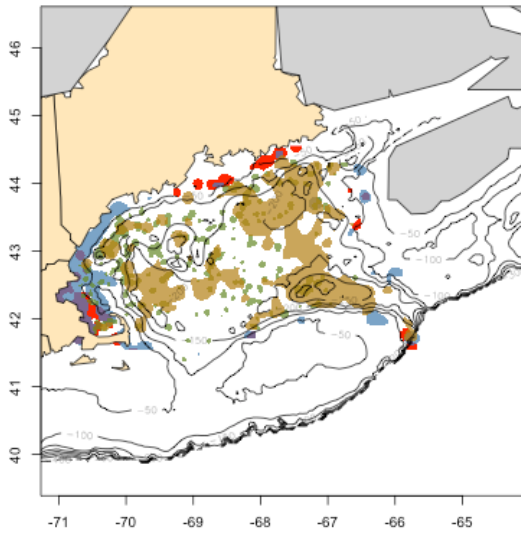

Fall

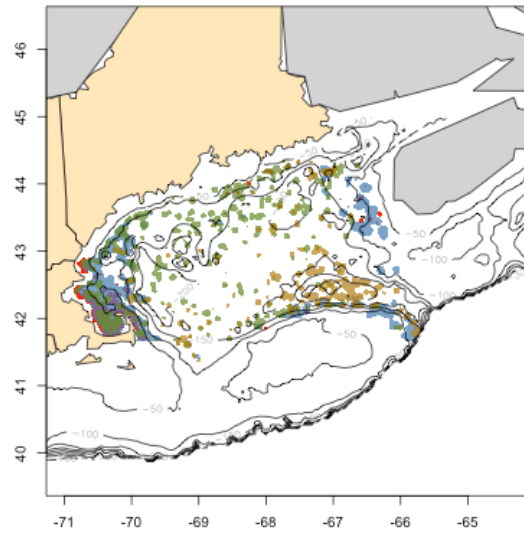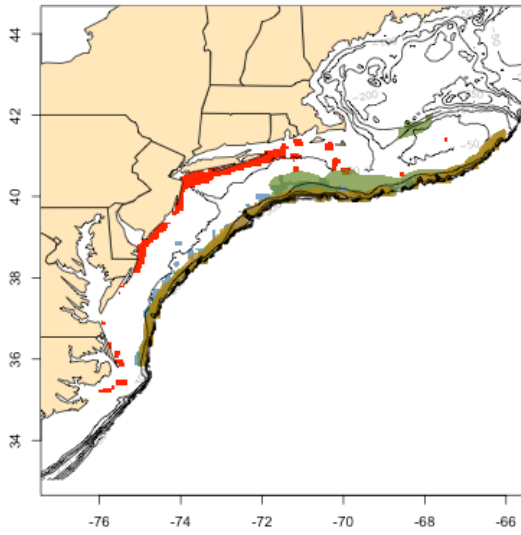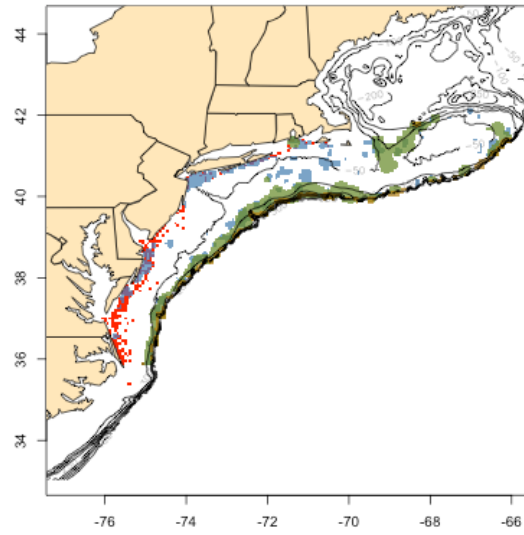

Supplement: S3 Fig — Red signifies clusters 1N and 1S, blue signifies clusters 2N and 2S, green signifies clusters 3N and 3S, and yellow signifies clusters 4N and 4S. The Gulf of Maine (northern NES) is shown in the top panels, and the Mid-Atlantic Bight/Georges Bank (southern NES) is shown in the bottom panels. Hotspots are based on kernel density values greater than one standard deviation above the mean. Black lines correspond to the 100 and 200m isobaths. (PDF) [file pone.0149220.s004.pdf]

**a.**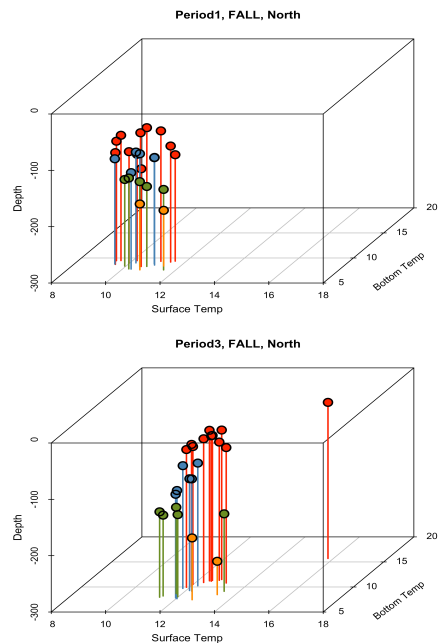**b.**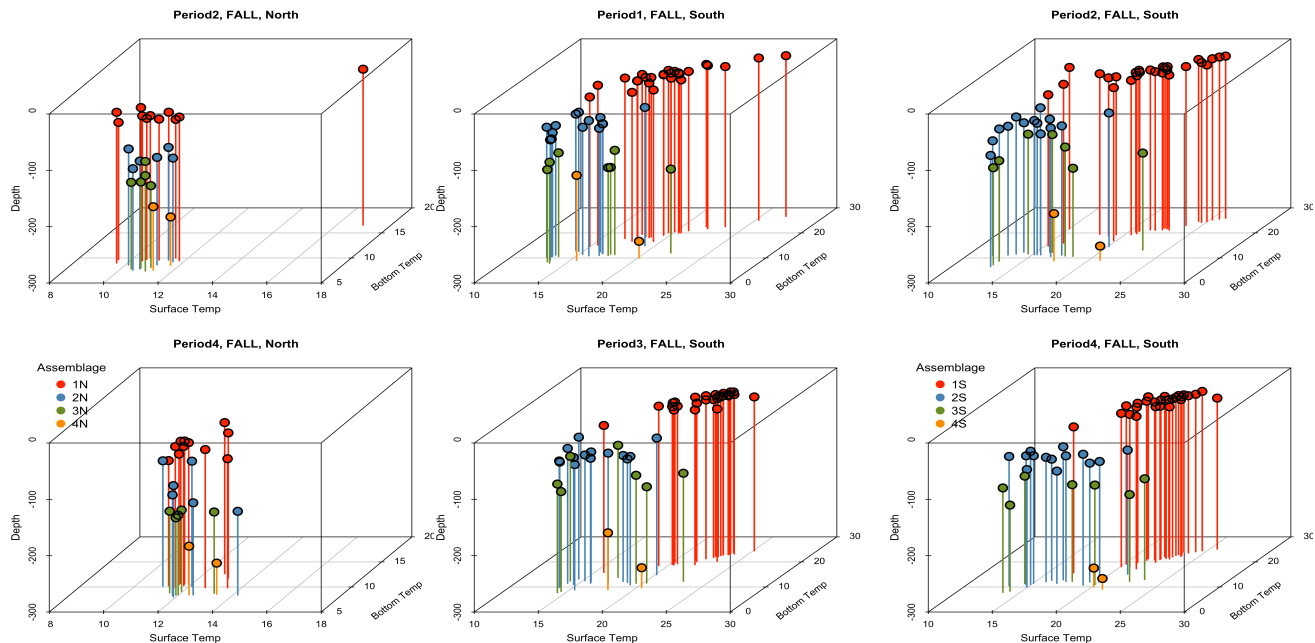**c.**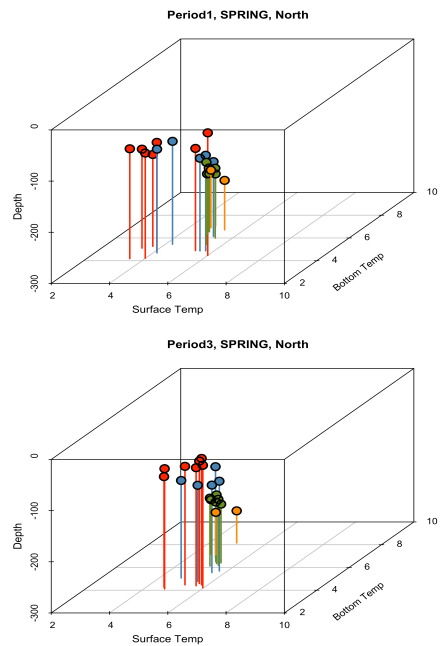**d.**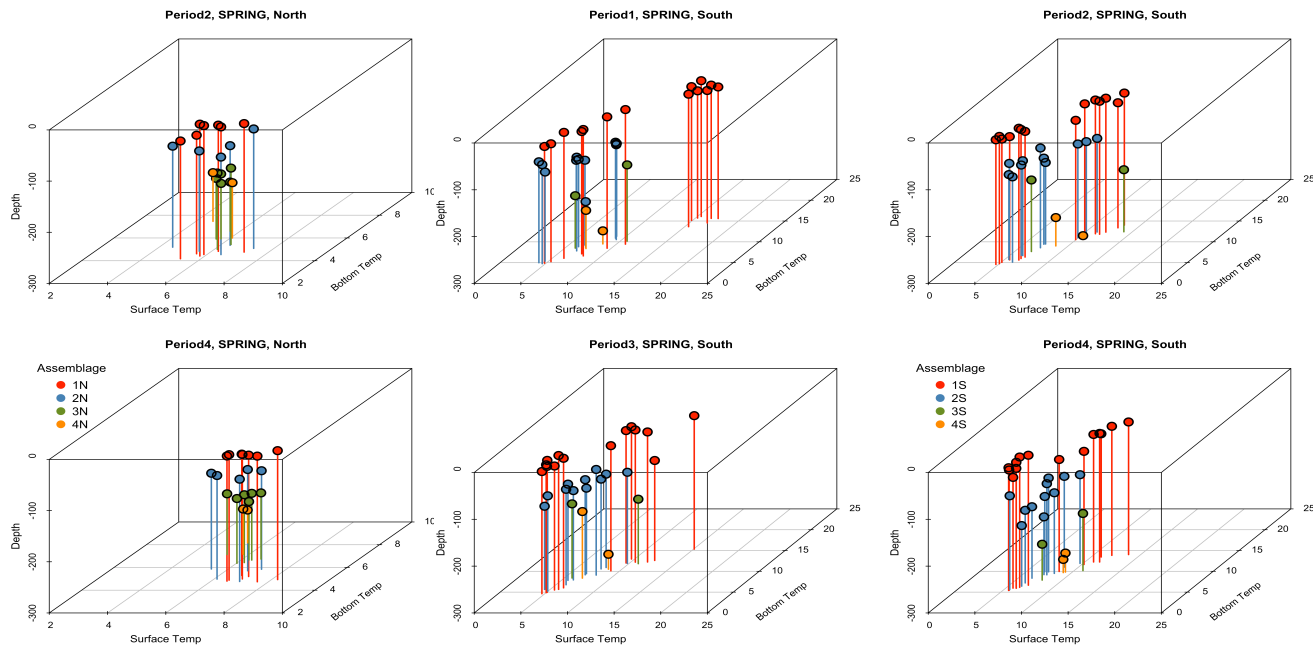

Supplement: S4 Fig — Assemblages are defined by surface temperature (x-axis), bottom temperature (y-axis), and depth (z-axis) in the Fall (A, B) and Spring (C, D) in the northern (A, C) and southern (B, D) NES. (PDF) [file pone.0149220.s005.pdf]

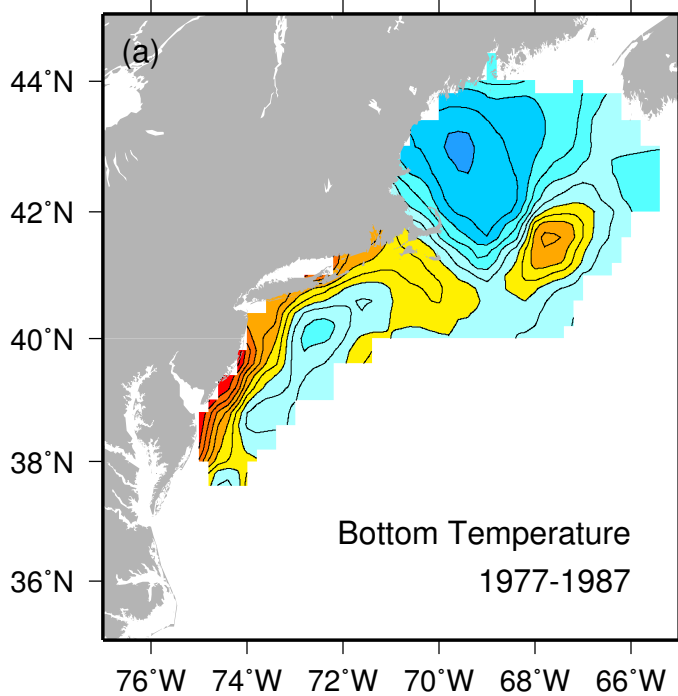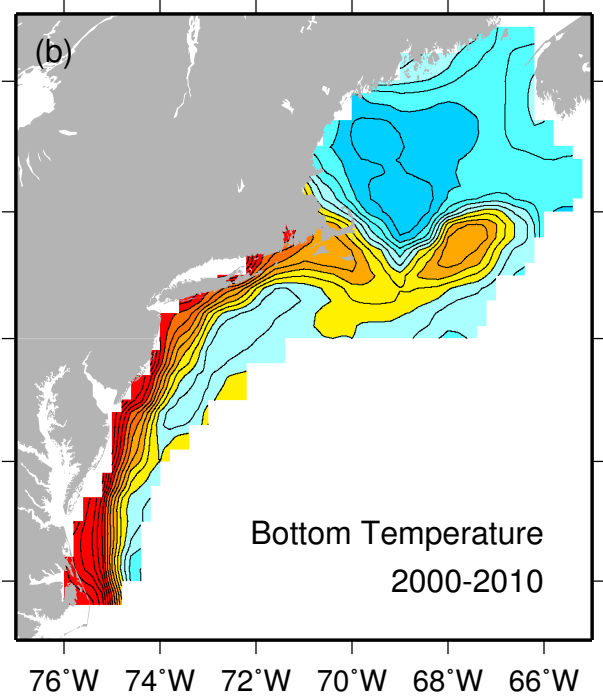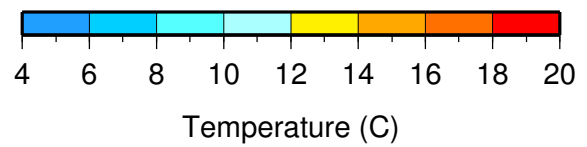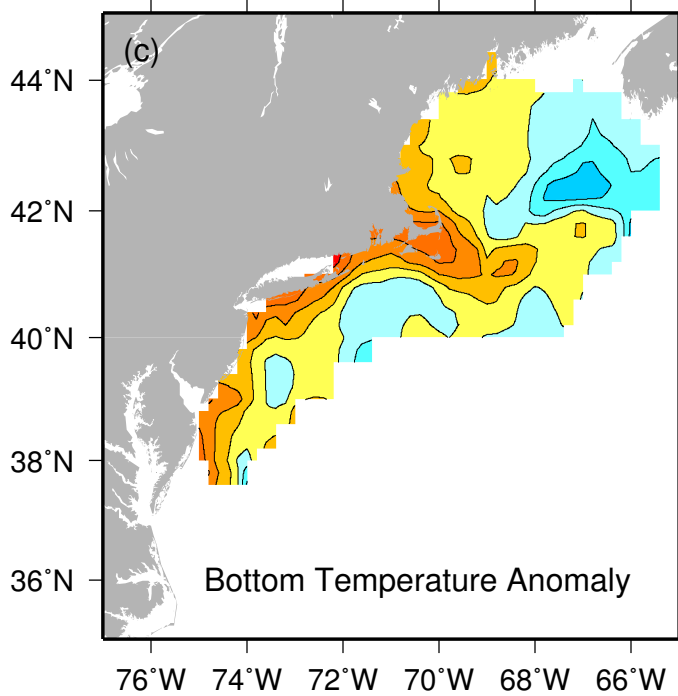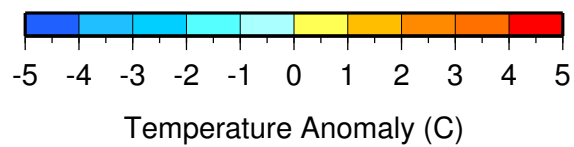

Supplement: S5 Fig — Comparison of average bottom temperature fields in the fall on the U.S. Northeast Shelf for an early part of the time series (1977–1987) and a later part of the time series 2000–2010. The bottom panel shows the difference field (late minus early). (PDF) [file pone.0149220.s006.pdf]

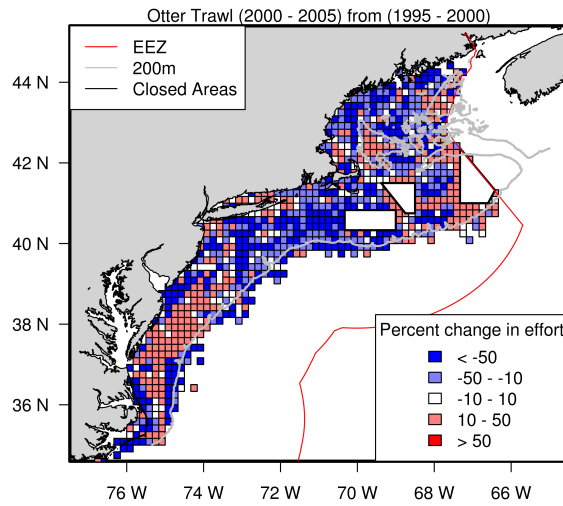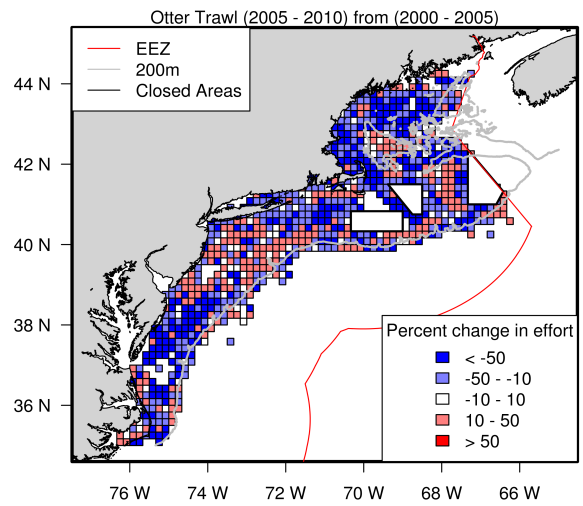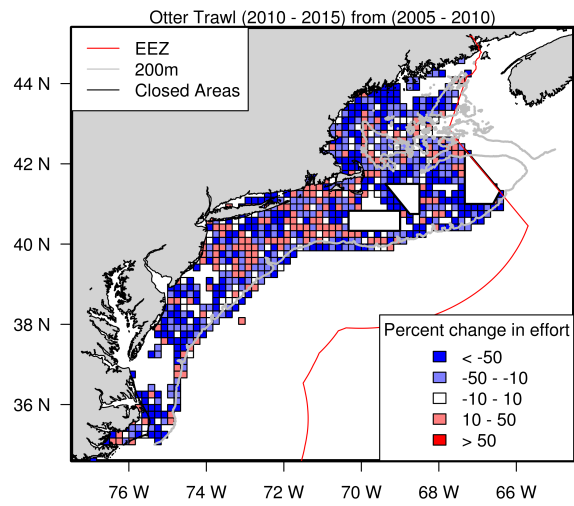

Supplement: S6 Fig — Blue (red) colors indicate a decrease (increase) in fishing effort between respective fishing periods. The EEZ is illustrated as a red line and the 200 m isobaths as a light grey line. Black lines illustrate the closed area boundaries. In general fishing effort has decreased in many area of the U.S. Northeast Shelf over the past two decades. (PDF) [file pone.0149220.s007.pdf]

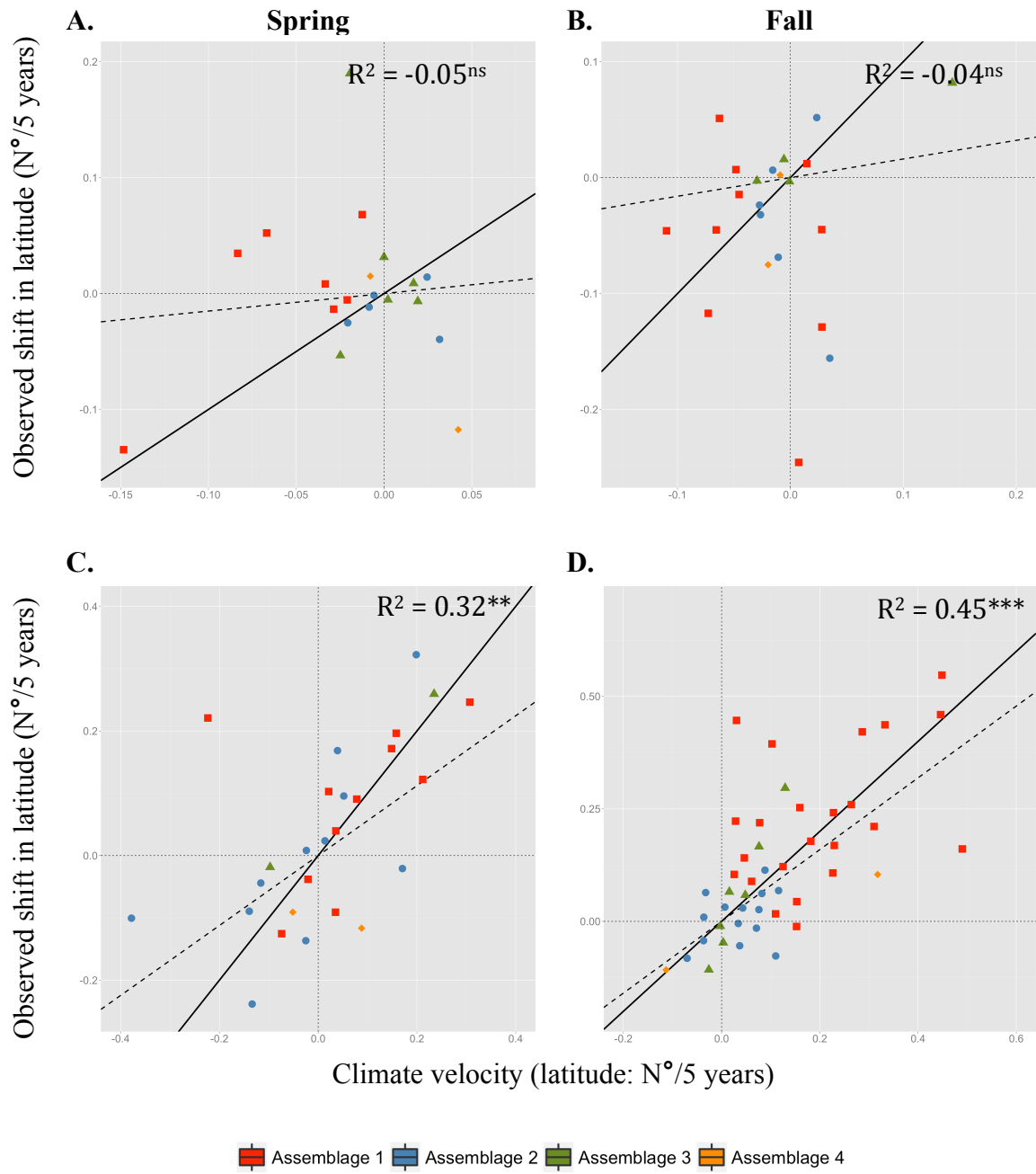

Supplement: S7 Fig — Slopes of observed versus predicted changes in latitude from truncated regressions for the Gulf of Maine (northern NES; a, b) and Mid-Atlantic Bight/Georges Bank (southern NES; c, d) northeast U.S. shelf sampled during spring (a, c) and fall (b, d) bottom trawl surveys. Colors correspond to clusters (red: cluster 1N or 1S; blue: cluster 2N or 2S; green: cluster 3N or 3S; yellow: cluster 4N or 4S). Significance is indicated by ‘ns’: not significant; ‘*’: p < 0.05; ‘**’: p < 0.01; ‘***’: p < 0.001. Significance is indicated by ‘ns’: not significant; ‘*’: p < 0.05; ‘**’: p < 0.01; ‘***’: p < 0.001. Solid black line is the 1:1 relationship and dashed black line corresponds to the linear model fit and provides a reference point for whether the assemblages are moving faster or slower relative to climate velocity with respect to latitude and depth. (PDF) [file pone.0149220.s008.pdf]
