# Supplementary material for: The Effects of Sub-Regional Climate Velocity on the Distribution and Spatial Extent of Marine Species Assemblages
Source: PLoS One. 2016 Feb 22;11(2):e0149220. doi: 10.1371/journal.pone.0149220 (PMC4762943; doi:10.1371/journal.pone.0149220)
Supplement: S1 Table — The presence of a ‘1’, ‘2’, ‘3’, or ‘4’ in the columns ‘Spring, South’, ‘Spring, North’, ‘Fall, South’, or ‘Fall, North’ indicates membership of a core species to a particular cluster. An ‘xx’ indicates that the species is present in a given region, but was not identified as a core species in any cluster and a blank entry indicates that the species is not present in either season in a given region. (DOCX) [file pone.0149220.s009.docx]

**S1 Table.** Description of the key species in the species clusters defined from the bottom trawl survey. The presence of a ‘1’, ‘2’, ‘3’, or ‘4’ in the columns ‘Spring, South’, ‘Spring, North’, ‘Fall, South’, or ‘Fall, North’ indicates membership of a core species to a particular cluster. An ‘xx’ indicates that the species is present in a given region, but was not identified as a core species in any cluster and a blank entry indicates that the species is not present in either season in a given region.

| **Species** | **Key** | **Trophic Level** | **Environment** | **Habitat** | **Spring, South** | **Spring, North** | **Fall, South** | **Fall, North** |
| --- | --- | --- | --- | --- | --- | --- | --- | --- |
| Acadian redfish (*Sebastes fasciatus*) | AcRed | 3.2 | Demersal | Found over rocky, mud, or clay bottoms in the Atlantic from the coast of Norway to Georges Bank. Off New England most common in the Gulf of Maine to depths of 975ft. |  | 3 |  | xx |
| Alewife (*Alosa pseudoharengus*) | Alewife | 3.5 | Pelagic-neritic | Movement of schooling adults apparently restricted to coastal areas proximal to natal estuaries. | xx | 2 | 2 | 1 |
| American lobster (*Homarus americanus*) | AmLob | 3.7 | Benthic | Found in rocky areas for shelter or in mud bottoms to burrow. Offshore populations most abundant along the edge of the continental shelf near underwater canyons. | 2 | xx | 3 | 1 |
| American plaice (*Hippoglossoides platessoides*) | AmPlai | 3.7 | Demersal | Adults are most abundant from 90 to 250m deep and bottom temperatures of -0.5 to 2.5°C. Live on soft bottoms. | 2 | 2 | 3 | 2 |
| American shad (*Alosa sapidissima*) | AmShad | 3.5 | Pelagic-neritic | Spend most of its life at sea, returning to freshwater streams to breed. Non-spawning adults are found in schools near the surface of continental shelf waters in spring, summer and fall; also found in brackish waters. | xx | xx | 2 | 2 |
| Atlantic cod (*Gadus morhua*) | AtlCod | 4.4 | Benthopelagic | Found from shoreline down to the continental shelf. Juveniles prefer shallow (less than 10-30m depth) sublittoral waters with complex habitats, such as seagrass beds, areas with gravel, rocks, or boulder, which provide protection from predators. Adults are usually found in deeper, colder waters. | xx | xx | 2 | xx |
| Atlantic croaker (*Micropogonias undulates*) | AtlCroak | 3.3 | Demersal | Adults occur over muddy/sandy bottoms in coastal waters and estuaries where nursery and feeding grounds are located. | 1 |  | 1 |  |
| Atlantic herring (*Clupea harengus*) | AtlHerr | 3.2 | Benthopelagic | Schools move between spawning and wintering grounds in coastal areas and feeding grounds in open water by following migration patterns learned from earlier year classes. Adults spend days in deeper water, but rise to shallow water at night. Light is an important factor in controlling vertical distribution. | xx | 1 | 2 | xx |
| Atlantic mackerel (*Scomber scombrus*) | AtlMac | 3.7 | Pelagic-neritic | Abundant in cold and temperate shelf areas, forms large schools near the surface. They overwinter in deeper waters but move closer to shore in spring when water temperatures range between 11° and 14°C. | xx | xx | xx | 1 |
| Banded drum (*Larimus fasciatus*) | BaDrum | 3.6 | Demersal | Occur usually over mud and sandy mud bottoms in coastal waters to about 60m, more rarely in estuaries. |  |  | 1 |  |
| Barndoor skate (*Dipturus laevis*) | Barndoor | 3.5 | Demersal | Found on mud bottoms as well as on sand and gravel. Occur from water's edge to 430m; broad temperature range; absent from shoal waters in south during warm months. | 2 | xx | 2 | 3 |
| Black sea bass (*Centropristis striata*) | BlSeaB | 4 | Reef-associated | Found around rock jetties and rocky bottoms in shallow water. | 2 |  | 1 |  |
| Blackbelly rosefish (*Helicolenus dactylopterus*) | BBRose | 3.8 | Bathydemersal | Soft bottom areas of the continental shelf and upper slope, depth range from 50 to 1100m. | 4 | 4 | 4 | 4 |
| Blueback herring (*Alosa aestivalis*) | BBHerring | 3.6 | Pelagic-neritic | Form schools and possibly wintering near the bottom and out from the coast, approaching the shore in the late spring. Spawn in brackish- or freshwaters of rivers. | 1 | xx | xx | 1 |
| Bluefish (*Pomatomus saltatrix*) | BlueF | 4.5 | Pelagic-oceanic | Occur in oceanic and coastal waters. Most common along surf beaches and rock headlands in clean, high-energy waters. Adults can also be found in estuaries and brackish water. | 1 |  | 1 |  |
| Bluntnose stingray (*Dasyatis say*) | Bluntnose | 3.5 | Demersal | Generally found near shore, to depths of 10m. |  |  | 1 |  |
| Bullnose stingray (*Myliobatis freminvillei*) | Bullnose | 3.2 | Benthopelagic | Found frequently in coastal waters to 10m, mainly in shallow estuaries. Capable of traveling long distances. |  |  | 1 |  |
| Butterfish (*Peprilus triacanthus*) | Butter | 4 | Benthopelagic | Forms large schools over the continental shelf, except during the winter months when it may descend to deeper water. | 2 |  | 2 | xx |
| Clearnose skate (*Raja eglanteria*) | Clearnose | 3.7 | Demersal | Found in saltier parts of estuaries and bays of 330m. Inhabit inshore areas. Prefers waters of 10°-21°C. | 1 |  | 1 |  |
| Cownose ray (*Rhinoptera bonasus*) | Cownose | 3.5 | Benthopelagic | Migratory oceanic species sometimes found near the coast. |  |  | 1 |  |
| Cravelle jack (*Caranx hippos*) | Cravelle | 3.5 | Reef-associated | Generally in neritic waters over the continental shelf. Adults ascend rivers. Juveniles abundant in brackish estuaries with muddy bottoms, near sandy beaches and on seagrass beds. |  |  | 1 |  |
| Cunner (*Tautogolabrus adspersus*) | Cunner | 3.5 | Reef-associated | Inhabits shallow, inshore waters, living on or near the bottom, often congregating in masses around wharves, wrecks and submerged seaweed. During winter they become torpid and remain inshore under rocks in shallow water. | xx | 1 | xx | xx |
| Cusk (*Brosme brosme*) | Cusk | 4 | Demersal | Found in small shoals on rough, rock, gravel, or pebble bottoms. Generally keeps far from the shore, near the bottom, mostly between 18 and 550m in the northwestern Atlantic. |  | 3 |  | xx |
| Fourspot flounder (*Hippoglossina oblonga*) | FSFlound | 4.2 | Demersal | Occurs in bays and sounds in the north range. | 2 | 2 | 2 | xx |
| Gulf Stream flounder (*Citharichthys arctifrons*) | GSFlound | 3.3 | Demersal | Occurs in deeper water. Rarely found in shallow water. | 2 |  | xx |  |
| Haddock (*Melanogrammus aeglefinus*) | Hadd | 4.1 | Demersal | Adults are found more commonly from 80 to 200m, over rock, sand, gravel or shells, usually at temperatures between 4° and 10°C. | xx | 2 | xx | 2 |
| Little skate (*Leucoraja erinacea*) | LiSkate | 3.6 | Demersal | Usually on sandy/gravelly bottoms from shoal waters to 90m. | 1 | 1 | xx | 1 |
| Longfin squid (*Doryteuthis pealeii*) | LongfinS | 3.5 | Benthopelagic | This is a neritic species found on sandy bottoms and along the water column. Spends winter at depths of 100 to 200m. | 2 | xx | xx | 1 |
| Longhorn sculpin (*Myoxocephalus octodecemspinosus*) | Longhorn | 3.5 | Demersal | Commonly found in harbors and shallow coastal waters. Move to deeper water in winter. | xx | 1 | 2 | xx |
| Monkfish (*Lophius americanus*) | Monkfish | 4.5 | Demersal | Inhabits continental shelf, occurring deeper in southern parts of range; tolerating a wide range of temperatures (0-21°C) and depths. | xx | 3 | 3 | 3 |
| Northern kingfish (*Menticirrhus saxatilis*) | NKing | 3.6 | Demersal | Occurs usually in shallow coastal waters over sand to sandy mud bottoms. Common in the surf zone and in estuaries. Juveniles may enter tidal rivers and creeks of low salinity. | xx |  | 1 |  |
| Northern puffer (*Sphoeroides maculatus*) | NPuff | 4 | Demersal | Inhabits bays, estuaries and protected coastal waters. |  |  | 1 |  |
| Northern sea robin (*Prionotus carolinus*) | NSeaR | 4.1 | Demersal | Found in estuaries and to the edge of the continental shelf. Occurs on sandy bottom. | xx |  | 1 |  |
| Pigfish (*Orthopristis chrysoptera*) | Pig | 3.4 | Demersal | Inhabits coastal waters, over sand and mud bottoms. |  |  | 1 |  |
| Pinfish (*Lagodon rhomboides*) | Pin | 3.6 | Demersal | Commonly found on vegetated bottoms, occasionally over rocky bottoms and in mangrove areas. Enters brackish water and even freshwaters. | 1 |  | 1 |  |
| Red hake (*Urophycis chuss*) | RedHake | 3.6 | Demersal | Found on soft muddy and sandy bottoms, but never on rocks, gravel or shells. Juveniles live along the coasts at shallow depths (4-6m); adults migrate to deeper waters, generally to between 110 and 130m, and in some instances, to over 550m. | 2 | 3 | 2 | 2 |
| Rosette skate (*Leucoraja garmani*) | Rosette | 3.6 | Reef-associated | Occur mostly on outer edge of continental shelf and upper part of continental slope. | 3 |  | 3 |  |
| Roughtail stingray (*Dasyatis centroura*) | Roughtail | 3.8 | Demersal | Found over sandy and muddy bottoms. Most common at a depth of 15–50m. |  |  | 1 |  |
| Scup (*Stenotomus chrysops*) | Scup | 3.9 | Demersal | Occurs in schools inshore in summer and offshore in winter. | xx |  | 1 | 1 |
| Sea raven (*Hemitripterus americanus*) | SeaRav | 4.5 | Demersal | Inhabit rocky or hard bottom and is a voracious eater. | xx | xx | 2 |  |
| Sea scallop (*Placopecten magellanicus*) | SeaScal | 2 | Benthic | Adults live clustered in beds on sandy or gravelly parts of ocean floor generally at depths of about 100 to 300ft on Georges Bank and in the Mid-Atlantic. Sea scallops can be found in shallower waters in Maine and Canada. | xx | xx | 2 | xx |
| Sharpnose shark (*Rhizoprionodon porosus*) | Shpnose | 3.9 | Reef-associated | Common in bays and estuaries often entering rivers. Also found in offshore waters at depths of about 500m, generally less than 100m. | xx |  | 1 |  |
| Shortfin squid (*Illex coindetii*) | Shortfin | 4 | Benthic | Inhabits muddy/sandy/detritic bottoms in middle and lower sub-littoral and upper bathyal zones in temperate latitudes. | xx |  | 3 | xx |
| Silver hake (*Merluccius bilinearis*) | SilverH | 4.3 | Demersal | Abundant on sandy grounds and strays into shallower waters. A voracious predator with cannibalistic habits. | xx | 3 | 2 | 3 |
| Smooth dogfish (*Mustelus canis*) | SmDog | 3.7 | Demersal | Found on continental and insular shelves and upper slopes, ranging from shallow inshore waters and the intertidal to 200m, occasionally down to 579m. | xx |  | 1 |  |
| Smooth skate (*Malacoraja senta*) | SmSkate | 3.5 | Bathydemersal | Confined to deeper water. Inhabits soft mud and clay bottoms of the deeper troughs and basins, sands and shells, gravel and pebbles of the offshore fishing banks. | xx | xx |  | 4 |
| Southern stingray (*Dasyatis americana*) | SSting | 3.5 | Reef-associated | Found on sandy bottoms, seagrass beds, lagoons and the reef face. Common in bays and estuaries. |  |  | 1 |  |
| Spiny butterfly ray (*Gymnura altavela*) | SpButter | 4.5 | Demersal | Occurs over sand and mud. |  |  | 1 |  |
| Spiny dogfish (*Squalus acanthias*) | SpDog | 4.3 | Benthopelagic | An inshore and offshore dogfish of the continental shelf and upper slopes. Usually near the bottom, but also in mid-water and at the surface. Often found in enclosed bays. | 2 | xx | 2 | 2 |
| Spot (*Leiostomus xanthurus*) | Spot | 3.9 | Demersal | Found usually over sandy or muddy bottoms in coastal waters to about 60m. Occurs in nursery and feeding grounds in river estuaries during summer and fall. | 1 |  | 1 |  |
| Striped bass (*Morone saxatilis*) | StBass | 4.5 | Demersal | Inhabit coastal waters and are commonly found in bays but may enter rivers in the spring to spawn. | 1 |  | 1 |  |
| Striped burrfish (*Chilomycterus schoepfii*) | StBurr | 3.3 | Reef-associated | Very common in seagrass beds in bays and coastal lagoons. Also found on shallow coastal reefs. |  |  | 1 |  |
| Striped sea robin (*Prionotus evolans*) | StSeaRob | 4.3 | Reef-associated | Occurs mainly on sand bottom from inshore estuaries to about 180m. Found occasionally over reefs. | xx |  | 1 |  |
| Summer flounder (*Paralichthys dentatus*) | SummFlou | 4.5 | Demersal | Adults prefer sandy substrate to burrow; broad range of lower and mid-estuary habitats: salt marsh/seagrass/sand flats. | xx |  | 1 |  |
| Tautog (*Tautoga onitis*) | Tautog | 3.3 | Reef-associated | Found close to shore on hard-bottom habitats, occasionally entering brackish water. | 1 |  | 1 |  |
| Thorney skate (*Amblyraja radiate*) | Thorney | 4 | Demersal | Cold temperate species found offshore on all kinds of bottoms, mainly sandy and muddy. In the northeast Atlantic common between 50-100m depth at temperatures of 2-5°C. | 2 | xx | 3 | xx |
| Weakfish (*Cynoscion regalis*) | Weak | 3.8 | Demersal | Occurs usually in shallow coastal waters over sand and sandy mud bottoms. | 1 |  | 1 |  |
| White Hake (*Urophycis tenuis*) | WhHake | 4.2 | Demersal | Found on soft, muddy bottoms of continental shelf/upper slope to 200m. Adults move inshore in northern Gulf of Maine in summer, disperse in fall, and deep in winter. | 3 | 4 | 3 | 3 |
| Windowpane flounder (*Scophthalmus aquosus*) | Window | 3.6 | Demersal | Occurs from shore to 45m, occasionally in deeper water. Found frequently in bays and estuaries. | 1 | 1 | 1 | 1 |
| Winter flounder (*Pseudopleuronectes americanus*) | WinFloun | 2.8 | Demersal | Adults inhabit soft muddy to moderately hard bottoms. | 1 | 1 | xx | 1 |
| Winter Skate (*Leucoraja ocellata*) | WinSkate | 4.4 | Demersal | Prefer sandy and gravelly bottoms in shoal water in the northern range, as deep as 90m in south. | 1 | xx | xx | 1 |
| Witch flounder (*Glyptocephalus cynoglossus*) | Witch | 3.1 | Demersal | Inhabits soft mud bottoms in fairly deep water | 4 | 3 | 4 | 3 |
| Wolffish (Anarhichas lupus) | Wolf | 3.2 | Demersal | Rocky/sandy/mud bottoms to 500m. Sedentary/solitary except for mating. Prefer rocky benthic habitat for shelter. | xx | 2 |  | 2 |
| Yellowtail flounder (*Limanda ferruginea*) | YtlFloun | 3.2 | Demersal | Adults inhabit sandy to muddy bottoms. Prefer depths of 40-80m at temperatures of 3-5°C. | xx | 1 | 2 | 1 |
